# Differential Impacts of the Head on *Platynereis dumerilii* Peripheral Circadian Rhythms

**DOI:** 10.3389/fphys.2019.00900

**Published:** 2019-07-11

**Authors:** Enrique Arboleda, Martin Zurl, Monika Waldherr, Kristin Tessmar-Raible

**Affiliations:** ^1^Max F. Perutz Laboratories, Vienna BioCenter, University of Vienna, Vienna, Austria; ^2^Research Platform “Rhythms of Life”, Vienna BioCenter, University of Vienna, Vienna, Austria

**Keywords:** marine, annelid, daily, rhythm, clock, chromatophores, transcription, locomotion

## Abstract

The marine bristle worm *Platynereis dumerilii* is a useful functional model system for the study of the circadian clock and its interplay with others, e.g., circalunar clocks. The focus has so far been on the worm’s head. However, behavioral and physiological cycles in other animals typically arise from the coordination of circadian clocks located in the brain and in peripheral tissues. Here, we focus on peripheral circadian rhythms and clocks, revisit and expand classical circadian work on the worm’s chromatophores, investigate locomotion as read-out and include molecular analyses. We establish that different pieces of the trunk exhibit synchronized, robust oscillations of core circadian clock genes. These circadian core clock transcripts are under strong control of the light-dark cycle, quickly losing synchronized oscillation under constant darkness, irrespective of the absence or presence of heads. Different wavelengths are differently effective in controlling the peripheral molecular synchronization. We have previously shown that locomotor activity is under circadian clock control. Here, we show that upon decapitation worms exhibit strongly reduced activity levels. While still following the light-dark cycle, locomotor rhythmicity under constant darkness is less clear. We also observe the rhythmicity of pigments in the worm’s individual chromatophores, confirming their circadian pattern. These size changes continue under constant darkness, but cannot be re-entrained by light upon decapitation. Our works thus provides the first basic characterization of the peripheral circadian clock of *P. dumerilii*. In the absence of the head, light is essential as a major synchronization cue for peripheral molecular and locomotor circadian rhythms, while circadian changes in chromatophore size can continue for several days in the absence of light/dark changes and the head. Thus, in Platynereis the dependence on the head depends on the type of peripheral rhythm studied. These data show that peripheral circadian rhythms and clocks should also be considered in “non-conventional” molecular model systems, i.e., outside *Drosophila melanogaster*, *Danio rerio*, and *Mus musculus*, and build a basic foundation for future investigations of interactions of clocks with different period lengths in marine organisms.

## Introduction

Extensive research focusing on drosophilids and mice showed that the daily behavioral, physiological and metabolic cycles in animals arise from coordination of central circadian clocks located in the brain and peripheral clocks present in multiple tissues (Richards and Gumz, 2012; Mure et al., 2018; Pilorz et al., 2018). In Drosophila, several peripheral tissues and appendages (e.g., Malpighian tubules, fat bodies, and antennae) have autonomous peripheral clocks that are directly entrained by environmental cycles independent of the central clock, while others, such as oenocytes, are regulated by the circadian clock located in the brain (Tomioka et al., 2012; Ito and Tomioka, 2016). The mammalian circadian system is highly hierarchically organized. The master central clock in the suprachiasmatic nucleus (SCN) of the brain (often referred to as a “conductor”) synchronizes internal clock timing to the environmental solar day by passing the information to the peripheral clocks via endocrine and systemic cues (Mohawk et al., 2012; Partch et al., 2014). These peripheral clocks also have self-sustained circadian oscillators, with the master clock coordinating their phase to prevent desynchronization among peripheral tissues, rather than acting as a pacemaker responsible for the periodicity of the cycling itself (Yoo et al., 2004). Besides being phase-controlled by the “SCN conductor,” several mammalian peripheral clocks (e.g., in liver and kidney) have been shown to directly respond to non-photic entrainment cues, like food or exercise (Tahara and Shibata, 2018).

For marine organisms, biorhythms of various period lengths, including circadian and circalunar, have been described across phyla, e.g., as changes in activity levels, coloration and reproductive cycles (reviewed in Tessmar-Raible et al., 2011; Last and Hendrick, 2014; Bulla et al., 2017; Raible et al., 2017). Where studied in detail, like in the marine bristle worm *Platynereis dumerilii* and the marine midge *Clunio marinus*, the light of sun and moon are known to serve as major entrainment cues (Hauenschild, 1960; Zantke et al., 2013; Kaiser et al., 2016). Over recent years, marine rhythms and their possible underlying clockworks have been receiving increasing attention, as the interplay of clocks and rhythms of different organisms is a crucial aspect for ecology (Schwartz et al., 2017).

Molecular data on rhythms and clocks in marine invertebrates have become increasingly available over the last decade, now including the bivalves *Mytilus californianus* (Connor and Gracey, 2011) and *Crassostrea gigas* (Perrigault and Tran, 2017), the sea slugs *Hermissenda crassicornis*, *Melibe leonina*, and *Tritonia diomedea* (Cook et al., 2018; Duback et al., 2018), the isopod *Eurydice pulchra* (Wilcockson et al., 2011; Zhang et al., 2013; O’Neill et al., 2015), the amphipod *Talitrus saltator* (Hoelters et al., 2016), the lobsters *Nephrops norvegicus* (Sbragaglia et al., 2015) and *Homarus americanus* (Christie et al., 2018), the mangrove cricket *Apteronemobius asahinai* (Takekata et al., 2012), the copepods *Calanus finmarchicus* (Häfker et al., 2017), and *Tigriopus californicus* (Nesbit and Christie, 2014), the Antarctic krill *Euphausia superba* (Mazzotta et al., 2010; Teschke et al., 2011; Pittà et al., 2013; Biscontin et al., 2017), the Northern krill *Meganyctiphanes norvegica* (Christie et al., 2018), the marine *midge C. marinus* (Kaiser and Heckel, 2012; Kaiser et al., 2016), and the marine polychaete *P. dumerilii* (Zantke et al., 2013; Schenk et al., 2019). On the marine vertebrate side, especially teleost fish species have been investigated (Park et al., 2007; Sánchez et al., 2010; Hur et al., 2011; Watanabe et al., 2012; Vera et al., 2013; Rhee et al., 2014; Toda et al., 2014; Mogi et al., 2015; Okano et al., 2017).

While most of the above mentioned species are difficult to maintain in the laboratory and to investigate at the level of molecular genetics, *P. dumerilii* is a particularly well-established laboratory model (Fischer and Dorresteijn, 2004; Fischer et al., 2010) for marine chronobiological research. It possesses interacting circadian and circalunar clocks, and complementing the molecular work, a detailed analysis of its circadian locomotor activities has been described for adult stages (Hauenschild, 1960; Zantke et al., 2013, 2014). Evidence of circadian activity also exists for young larval stages within the first days of their development (Tosches et al., 2014). Similar to the isopod *E. pulchra* (Wilcockson et al., 2011; Zhang et al., 2013), *P. dumerilii* also exhibits a circadian rhythm in its body pigmentation (Fischer, 1965; Röseler, 1969, 1970). This rhythm in pigment cell extension versus contraction was described as a segment-autonomous process (Fischer, 1965; Röseler, 1969, 1970), indicating the presence of autonomous peripheral circadian oscillators.

As *P. dumerilii* beheaded individuals survive well for up to 2 weeks (Hofmann, 1976), this feature can be used to study living animals in the absence of its circadian brain clocks. Moreover, *P. dumerilii* has primitive morphological and genetic features, and is hence viewed as evolutionarily slowly evolving (Tessmar-Raible and Arendt, 2003), a feature which is particularly interesting for understanding the ancestral features of different clocks and rhythms, as well as in the light of the- certainly debated- hypothesis that vertebrates originated from a polychaete-like animal (Last and Hendrick, 2014).

The work presented here is the first detailed characterization of several *P. dumerilii* peripheral circadian rhythms and clocks, covering analyses of transcript level changes of core circadian clock genes, as well as body pigmentation and locomotor activity.

## Materials and Methods

### Animal Cultures

Animals were maintained under controlled temperature and on 16:8 h light-dark (LD) or dark-dark (DD) cycles as previously described (Schenk et al., 2019). Sampling points are presented either as zeitgeber time (ZT) for LD conditions or circadian time (CT) for constant darkness (DD). ZT0 is defined as the time of light ON, CT0 defined as the time the light would go ON under DD conditions. All animal work was conducted according to Austrian and European guidelines for animal research.

### Worm Decapitation

Animals were anesthetized by adding a few drops of 1 M MgCl_2_ in the seawater until they stopped moving. They were carefully placed on a microscope slide under a binocular dissecting microscope, decapitated, and transferred to fresh seawater again. Decapitation was done with sterile surgical blades cutting on the first segments below the pharyngeal region in order to collect only the posterior part of the body (i.e., the trunk). They were consistently performed between ZT 13.5 and ZT14. For re-entrainment, ZT refers to the actual ZT of the new LD regime. For head samples, the region containing the pharynx and the posterior end of the head was removed (see Zantke et al., 2013).

### Constant Darkness (DD) Condition

Groups of animals used for DD experiments were collected between ZT 13.5 and ZT14 on individual plastic cups with seawater, wrapped in aluminum paper and placed inside a custom-made dark box in a temperature-controlled environment. After the proper acclimation time (1–5 days depending on the experiment) a plastic cup was taken out at a given CT and animals were used for RNA extraction/RT-qPCR or pigmentation quantification. This method ensured the avoidance of risking a premature exposure to light to the remaining samples in the dark box. Further animal handling (e.g., for decapitation or chromatophore visualization) was done under dim light and as fast as possible.

### Circadian Re-entrainment Under White, Blue, and Red Light Conditions

When testing for circadian re-entrainment (i.e., an inverted light cycle) under white, blue, or red light, animals were exposed to the new conditions for 7 days before sampling. Light spectra and intensities of white, blue, and red LEDs (ProfiLuxSimu-L from GHL advanced technology gmbh, Germany) used for circadian re-entrainment were measured using an ILT950 spectrometer (International Light Technologies Inc., Peabody, United States). Special care was taken to account for the standard conditions, where the worms were housed, i.e., 22 cm away from light source and with a transparent plastic lid positioned between the spectrometer and the light source. Measured light intensities for white, red, and blue lights were 8.2 × 10^13^ photons/cm^2^/s, 3.8 × 10^13^ photons/cm^2^/s, and 2.4 × 10^13^ photons/cm^2^/s, respectively (for spectra, see Supplementary Figure 1).

### Total RNA Extraction and RT-qPCR

Total RNA was extracted from heads or trunks (i.e., decapitated animals) using the RNeasy Mini Kit (QIAGEN). In the case of heads, each biological replica consisted of 4–5 heads (to obtain sufficient amounts of RNA), while for trunks each replica consisted of a single decapitated animal. Reverse transcription was carried out using 0.4 μg of total RNA as template (QuantiTect Reverse Transcription kit, QIAGEN). RT-qPCR analyses were performed using a Step-One-Plus cycler. The expression of each test gene was normalized by the amount of the internal control gene cdc5. The relative expression was calculated using the formula 1/2^ΔCt^. Primers and PCR program used are listed in Zantke et al. (2013).

### Chromatophore Size

Three consecutive segments located toward the middle of the body were selected on each animal to evaluate changes in chromatophore size. In order to precisely re-identify the same segments over the course of the experiments, animals were anesthetized with MgCl_2_ and a parapodium, two segments away from the region of interest, was removed with a sterile surgical blade. When required, one animal at a time was placed on a glass cover without water and extended carefully. An epifluorescence stereoscope (Zeiss Lumar) with a 488 nm laser and a FITC filter was used to take pictures making sure to always use the same magnification across animals and sampling points. Animals were placed again in seawater until the next sampling point. Image analysis was done using Adobe Photoshop. On fluorescent images, the RGB channels red and blue were lowered to zero, and the three segments of interest were extracted by erasing the unwanted area (chromatophores on each segment have a specific pattern, which makes their visual identification easier). A new layer was generated by using the magic wand tool to single out the bright green chromatophores from the background fluorescence. Using the image’s histogram, the number of colored pixels was used as a proxy of chromatophore size. Animals had between 20 and 40 chromatophores along the three segments, but no effort was done to quantify size of individual chromatophores. Instead, the sum of all the chromatophores of interest were used as chromatophore size value at each time point. Absolute pixel number was expressed as a percentage of the maximum value for each animal across all time points of the experiment [i.e., x_i_/(Max{x_i_,…,x_j_}^*^100)]. Average among biological replica and SEM were further calculated.

### Locomotor Activity Assay

Immature worms of comparable size were starved for 3 days before the start of the assay. After decapitation, worms were placed in individual hemispherical concave wells (diameter = 35 mm, depth = 15 mm) of a custom-made 36-well clear plastic plate [as described in Ayers et al. (2018)]. Intact worms were also treated with MgCl_2_ for 5 min prior to locomotor recording to ensure proper comparisons to decapitated worms. Video recording of worm’s behavior over several days was accomplished as described previously (Zantke et al., 2013), using an infrared (λ = 990 nm) LED array (Roschwege GmbH) illuminating the behavioral chamber and an infrared high-pass filter restricting the video camera. Worms were recorded over 4 LD cycles (16 h light/8 h darkness), followed by 3 days under constant darkness. White light was generated by custom made LEDs (Marine Breeding Systems, St. Gallen, Switzerland) with the intensity set to 5,2 × 10^14^ photons/cm^2^/s at the place were worms were housed (for light spectrum see Supplementary Figure 1). Trajectories of locomotor activity of individual worms were deduced from the video recordings by an automated tracking software developed by LoopBio gmbh^1^ (Vinoth Babu Veedin Rajan et al., unpublished). Locomotor activity trajectories reflect the distance moved of each worm’s center point across 6 min time bins. Activity data was plotted as double-plotted actograms using the ActogramJ plugin for Fiji (Schmid et al., 2011). For primary data see Supplementary Data 2.

### Statistical Analyses

The main statistical analyses were performed using either the data analysis plug-in in Microsoft Excel using an alpha value of 0.05 (molecular and chromatophore data) or GraphPad Prism (locomotor activity data). For changes in chromatophore size, each two-sample two-tailed student’s *t*-test was preceded by an *F*-test to check if the variances of the two groups were equal or not. In the cases were the same animals were used over time (i.e., repeated measures), a paired two-tailed Student’s *t*-test was used. To test if transcriptional changes in gene expression over time oscillated to a statistically significant difference (i.e., the curve was not statistically linear), fold change data was analyzed for each sampling point using single factor ANOVA. In order to ease the logistic process of analyzing a considerable amount of data, RNA samples and chromatophore size images were not analyzed blindly but chronologically as experiments were being performed. Two-way ANOVA and *post hoc* tests were performed in R v3.5.1 (R Core Team, 2018) with the package emmeans. All data sets were checked if the assumption of homogeneity of variances and normality were met. In the case of repeated measures ANOVA, GraphPad Prism software was used; sphericity was not assumed and the method of Greenhouse and Geisser was used to adjust the results (see Supplementary Data 1 for details on two-way ANOVA analyses). Statistical differences in locomotor activity across treatments were estimated using repeated measures ANOVA (GraphPad Prism) followed by Sidak’s multiple comparison test. To identify the free-running period length of intact worms under DD conditions Lomb-Scargle periodogram analysis was done using the ActogramJ plugin for Fiji (Schmid et al., 2011).

### Availability of Data and Material

All sequence resources referred to here were already published previously and submitted to public databases. All other data that support the findings of this study are available from the corresponding author upon reasonable request.

## Results

### *Platynereis dumerilii* Peripheral Circadian Clock Gene Transcripts Quickly Desynchronize Under Complete Darkness

**FIGURE 1:**
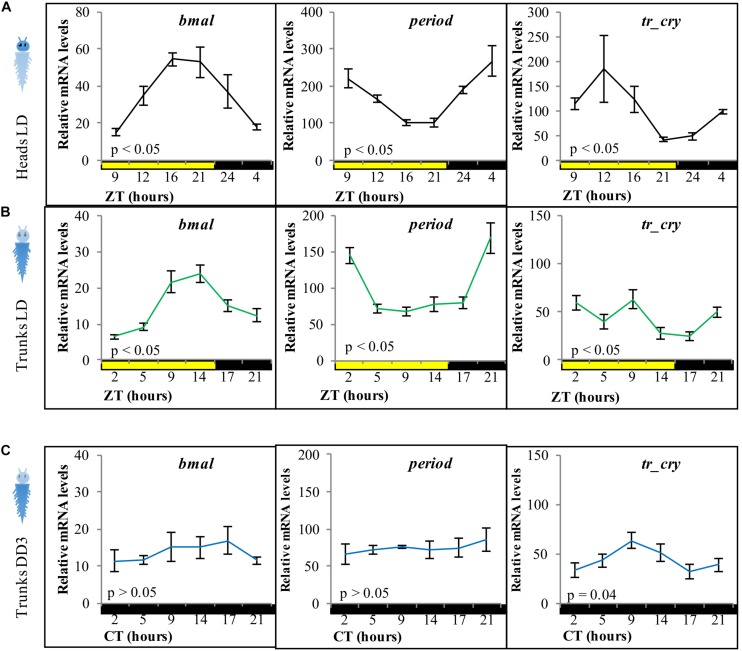
Relative transcript levels of *bmal, period*, and *tr-cry* in panel **(A)** heads under LD conditions, **(B)** trunks under LD conditions, and **(C)** trunks in DD conditions for 3 days on intact animals (i.e., not decapitated). ZT, zeitgeber time; CT, circadian time. *p*-Value estimated on a single factor ANOVA with *n* = 6, 15, and 11 for panels **(A–C)**, respectively (alpha = 0.05). Error bars denote SEM.

**FIGURE 2:**
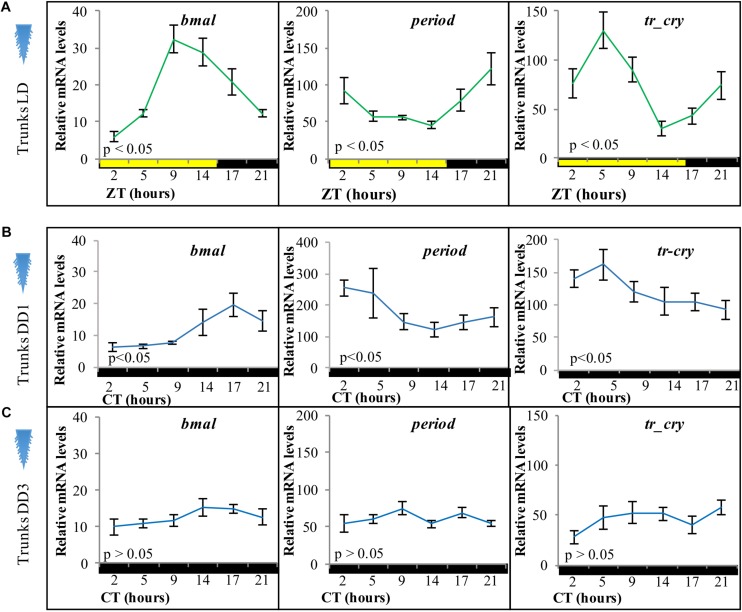
Relative transcript levels of **(A)** *bmal*, **(B)** *period*, and **(C)** *tr-cry* in trunks of decapitated animals placed under **(A)** LD, **(B)** DD conditions for 1 day, and **(C)** DD conditions for 3 days. ZT, zeitgeber time; CT, circadian time. *p*-Value estimated on a single factor ANOVA with *n* = 6 on each condition (alpha = 0.05). Error bars denote SEM.

**FIGURE 3:**
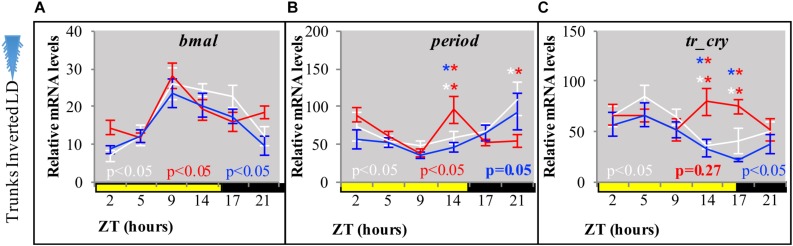
Relative transcript levels of **(A)** *bmal*, **(B)** *period*, and **(C)** *tr-cry* in trunks of decapitated animals placed under inverted LD conditions for 7 days with either white, blue, or red “light color” (for light spectra and intensity see Supplementary Figure 1). ZT, zeitgeber time. CT, circadian time. *p*-Value estimated on a single factor ANOVA with (alpha = 0.05). Error bars: SEM. Asterisks: ZTs, where statistically significant differences between light treatments were found after a two-way ANOVA and *post hoc* pairwise comparisons of estimated marginal means, Tukey adjusted with alpha = 0.05 (see also Supplementary Data 1).

As a starting point, we aimed to test for the presence of peripheral clocks in the body of *P. dumerilii* adults. Based on the previously described and tested molecular components of the core circadian oscillator in *P. dumerilii* heads (Zantke et al., 2013), we analyzed the daily transcriptional fluctuation of *bmal*, *period* and *tr-cry* as representative components of the circadian clock by RT-qPCR (Figure 1). Daily fluctuation of *bmal, period*, *tr-cry*, *timeless*, *clock*, and *pdp1* transcripts in the trunk are overall similar to those in the head [Figures 1A,B, for *timeless*, *clock*, and *pdp1*, see Supplementary Figures 2A–C, compare also with (Zantke et al., 2013)], although overall relative mRNA levels were always significantly higher in heads than in trunks. In the case of *period*, there was also a significant difference in overall transcript levels between trunks under LD and DD conditions, with higher levels at LD (pairwise comparisons of estimated marginal means, Tukey adjusted with alpha = 0.05) (see Supplementary Data 1 referring to Figure 1). Results were similar irrespective of the segment positions used for analyses, i.e., whole trunk (Figure 1), last 5–7 segments of the body or the adjacent 5–7 segments toward the anterior part of the animal (Supplementary Figures 2D–F) produced consistent results. *Pdu-tr-cry* appears to be the gene that deviates most in this comparison. We attribute this difference to a higher variability of the transcript level synchronization in the trunk given that in most cases it corresponds to the oscillations observed in the head. Alternatively, the presence of the head leads to a shift in the *tr-cry* curve maxima, as the curves for *tr-cry* consistently differ when the head is present (compare Figures 1A,B, 2A,B, 3C and Supplementary Figure 2F, and see also Zantke et al., 2013 for heads, Tosches et al., 2014 for whole larvae). A two-way ANOVA on ZT and condition (i.e., LD Heads and LD Trunks) was carried out to further analyze *tr-cry* transcript changes. There was a statistically significant effect of ZT, condition and, most importantly, interaction between the ZT and the condition on relative mRNA levels [*F*(5,95) = 5.3837, *p* = 0.0002]. A *post hoc* pairwise Tukey analysis for the interaction shows that the low relative mRNA levels at ZT5 on trunks under LD conditions is the main difference between these two conditions (see Supplementary Data 1).

We next tested if the peripheral circadian clock transcript oscillations would continue synchronously under constant darkness for 3 days. All tested transcripts dampened strongly, with *tr-cry* still exhibiting weak significant changes across CT (Figure 1C).

### Light Signals Are Sufficient to Maintain Circadian Clock Transcripts in the Trunk, Independent of the Head

To analyze if the peripheral circadian clock transcript changes in the worm’s body were dependent on the circadian clock in its head, we took advantage of the fact that bodies of decapitated animals survive in seawater for about 2 weeks (Hofmann, 1976). Adult animals were decapitated and placed under standard LD cycles before RNA extraction. Relative transcript levels of *bmal*, *period*, and *tr-cry* exhibited overall similar levels and continued a clear diel cycling of expression for at least 3 days in trunks of decapitated animals (Figure 2A), indicating that the peripheral circadian clock gene expression continues to synchronously run even in the absence of the brain circadian clock. We then tested core circadian clock transcript changes in trunks of decapitated animals under constant darkness. On the first day of DD (DD1) trunks continue to show a significant oscillation over time in all three genes (Figure 2B). However, after 3 days in complete darkness (DD3), relative mRNA levels dampen and no significant changes were detectable for any of the three genes (Figure 2C). We also tested for overall relative mRNA levels of expression of each gene under each condition (pairwise comparisons of estimated marginal means, Tukey adjusted with alpha = 0.05, see Supplementary Data 1 for Figure 2). In the trunk *bmal* overall levels were higher in LD than in DD1 and DD3 conditions (which in turn were statistically similar among them), while overall *period* levels were higher in DD1 compared to DD3 and LD conditions (*statistically similar among them*). For *tr-cry*, although LD is also clearly still higher, differences in overall levels on all three conditions are statistically significant (see Supplementary Data 1 referring to Figure 2). These differences possibly reflect differences in the transcriptional activation and repression during the different light conditions, e.g., it is noteworthy that under DD1 levels of the transcriptional suppressor *per* get significantly higher, while the transcript levels of the corresponding “downstream” transcriptional activator *bmal* are lowered under DD1, as would be expected if more *per* is present and translated to protein.

By placing decapitated animals on an inverted light cycle, we next tested for the capacity of peripheral clocks to be re-synchronized by light, again using the transcript oscillations of *bmal*, *per*, and *tr-cry* as readout. Cycling of *bmal* and *per* was re-entrained to the inverted cycle when exposed to white, red or blue light (Figures 3A,B, for spectra and intensity see Supplementary Figure 1). *tr-cry* transcript oscillations differed from this, in that white and blue light could re-entrain its peripheral oscillations as in the case of *bmal* and *per*, whereas red light did not (Figure 3C). To better understand these findings, a two-way ANOVA to simultaneously examine the effect of light treatment (i.e., light color = wavelength) and ZT was performed for each gene (see Supplementary Data 1). In the case of *bmal*, ZT [*F*(5,146) = 12.6907, *p* = 3.0399e^–10^] had a significant effect; with ZT5 and ZT21 having the same levels of expression. A similar result was found for *per*, but with the important addition of a significant interaction between ZT and the light condition (*F*(10,144) = 2.7811, *p* = 0.0036), indicating that the relationship of ZT and relative mRNA levels is dependent on the type of light. A *post hoc* pairwise Tukey analysis for the interaction indicates that such dependence is due to a specific variation on relative mRNA levels of *per* on tails under red light at ZT14 and ZT21. In the case of *tr-cry*, the situation is more complex. The analysis shows an effect of ZT, Light and a significant interaction between the two [*F*(10,144) = 2.1109, *p* = 0.0271] on relative mRNA levels. The *post hoc* pairwise analysis of light treatments (i.e., colors) over each ZT shows statistical differences for red light at ZT14 and ZT21, with significantly higher relative mRNA values compared to white or blue light at the same ZTs (Supplementary Data 1 referring to Figure 3). These punctual higher relative mRNA levels are the clearest evidence for the mentioned lack of re-entrainment of *tr-cry* peripheral oscillations by red light. Overall, these results demonstrate that circadian clock transcripts in peripheral tissues can directly respond to changes in the light cycle, independently of the head.

### Chromatophore Size Follows a Circadian Pattern and Free-Runs Under Constant Darkness

In order to assess how our findings on core circadian clock transcripts oscillations might relate to physiology and behavior, we investigated possible outputs of peripheral circadian clocks, starting with changes in chromatophore size in the trunk. Chromatophores are located along the dorsal part of the segmented body of *P. dumerilii*. Based on light microscopy analyses it had previously been shown that the worm’s chromatophores exhibit a segment-autonomous, diel contraction-expansion rhythm with increasing size during the day and decreasing size at night (Fischer, 1965; Röseler, 1969, 1970).

**FIGURE 4:**
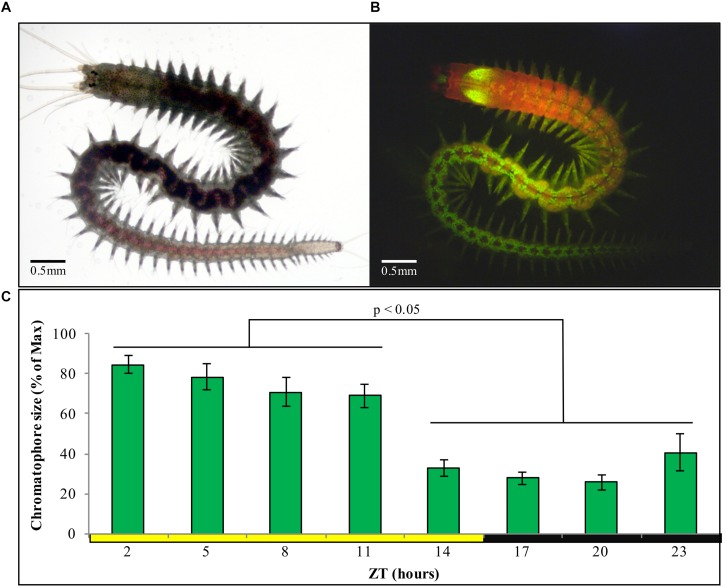
**(A)** Sexually immature *P. dumerilii* adult under standard light microscopy. **(B)** Same individual as in panel **(A)**, now autofluorescent under 488 nm light and a FITC filter, with chromatophores clearly visible as bright green circles (prominent green autofluorescence of the jaws can also be see in the anterior end). **(C)** Average chromatophore size based on autofluorescence (see materials and methods) of the same group of individuals followed over a 24 h period (*n* = 15) under standard LD conditions. Pairwise Student’s *t*-tests (preceded by *F*-tests) were performed comparing each of the ZT h. Individual averages from ZT2 to ZT5 and ZT14 to ZT23 were statistically similar within them but not between them in all permutations possible (alpha = 0.05). Error bars denote SEM.

We first aimed at identifying a possibility to automatize the recording of chromatophore changes. We found that chromatophores exhibit a well-detectable autofluorescence under 488 nm light (Figures 4A,B), which can be used for automatic detection by any image software. In order to characterize the pattern of contraction/expansion of the chromatophores, animals were photographed every 3 h over the course of 24 h using a fluorescence microscope. We found a clear circadian pattern with higher chromatophore expansion between ZT2 and ZT11 (Figure 4C), and a sharp drop on chromatophore size from ZT11 to ZT14, before lights go off and the subjective night period starts, already suggestive of an autonomous clock-driven process and not a direct light response, again consistent with previous observations (Hempelmann, 1939; Fischer, 1965).

**FIGURE 5:**
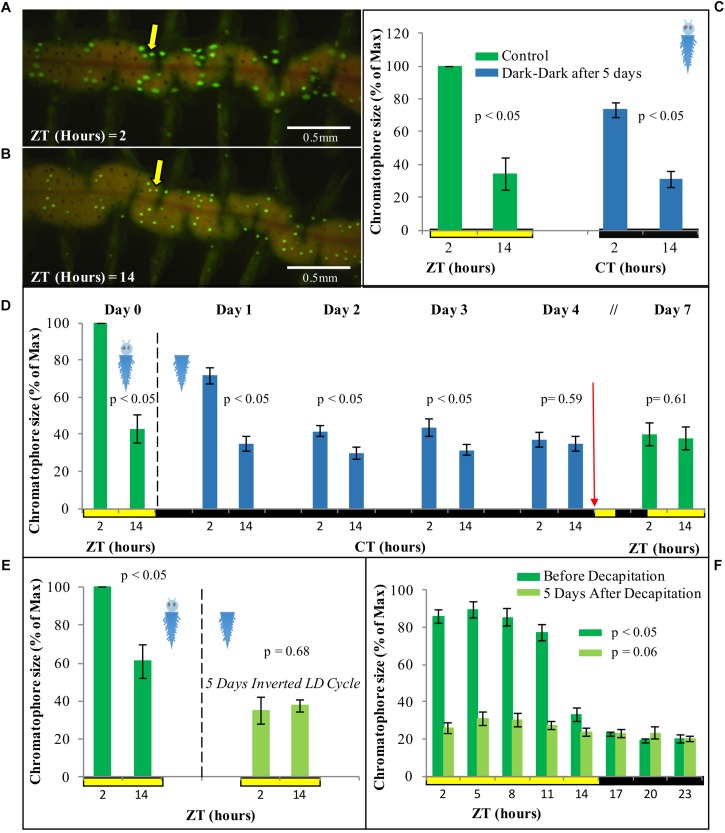
Fluorescent microscopy images of chromatophore size difference between **(A)** ZT2 and **(B)** ZT14 under standard LD conditions. **(C)** Average chromatophore size at ZT/CT 2 and ZT/CT 14 before and after 5 days under DD conditions (*n* = 4). **(D)** Average chromatophore size at ZT/CT 2 and ZT/CT 14 over seven consecutive days. Dashed line indicates decapitation and placement in DD conditions. Red arrow indicates re-placement under LD conditions (*n* = 6) (see Supplementary Figure 3 for individual replicas). **(E)** Average chromatophore size at ZT2 and ZT14 of individuals before and 5 days after decapitation and placement on an inverted LD cycle (*n* = 6). **(F)** Average chromatophore size, over a 24 h period, of individuals before and 5 days after decapitation (*n* = 10). For panel **(C)** pairwise Student’s *t*-tests (preceded by *F*-tests) were performed comparing ZT/CT 2 and ZT/CT 14 at each sampling day. For panels **(D,E)**, the pairwise student’s *t*-tests were paired (i.e., repeated sampling). For panel **(F)**, *p*-Value was estimated on a single factor ANOVA. Error bars denote SEM and alpha = 0.05 in all cases.

We next focused on sampling points corresponding to ZT/CT2 and ZT/CT14, during which a ∼60% drop in chromatophore size was evident (Figures 4C, 5A,B), and used these two time points as a reference for the study of circadian cycling of pigmentation over multiple days. Chromatophores expansion/contraction continued to cycle in animals placed under constant darkness for five consecutive days (Figure 5C).

For evidence that the light used for measuring chromophore size is not causing re-entrainment in this case, see below.

### Circadian Pattern of Chromatophore Size Free-Runs, but Cannot Be Re-entrained by Light in Decapitated Animals

In order to assess if the regulation of the cycling on chromatophore size is governed by peripheral clocks, decapitated animals were used. The same animals were photographed at ZT2 and ZT14 prior to decapitation as a starting reference point. Following decapitation, worms were placed in constant darkness for 4 days, and subsequently exposed again to a normal LD cycle for additional 3 days. Chromatophores sizes were registered along the experiment from day 0 to day 4, and again on day 7 to test for possible re-entrainment. Upon decapitation, animals under DD conditions initially continue to exhibit clear rhythms of chromatophore size changes (Figure 5D, for individual replicas see Supplementary Figure 3). Starting with the second day in DD, the rhythm will, however, start to dampen and become statistically non-significant by day 4 (Figure 5D). Subsequent exposure to a normal LD cycle did not lead to a re-synchronization of the chromatophore rhythm (Figure 5D). Consistently, cycling of chromatophore size does not get re-entrained on decapitated animals under an inverted LD cycle applied for 5 days (Figure 5E). A two-way repeated measurement ANOVA and *post hoc* pairwise analyses, confirmed a significant interaction between CT (i.e., time of subjective day) and the different days in DD [*F*(3,15) = 13.34, *p* = 0.0050], indicating that the difference between CT2 and CT14 gets significantly smaller over time from DD1 to DD4 (see Supplementary Data 1 referring to Figure 3).

In order to rule out that we may have missed phase-shifts on decapitated animals due to the exposure to the 488 nm light during the measurement procedure (due to too low sampling frequency), we also performed a more densely spaced 24 h sampling on day 5 in LD (post decapitation). This experiment confirmed our interpretation of a dampening of the chromatophore size rhythm and inability to re-entrain in the absence of the head (Figure 5F). All together, these results suggest a circadian pattern of chromatophore size governed by a peripheral clock, which, however, requires the head to maintain extended synchronization and for re-entrainment by light.

### Circadian Locomotor Activity Follows the Light-Dark Cycle, but Does Not Free-Run Under Constant Darkness in Decapitated Worms

**FIGURE 6:**
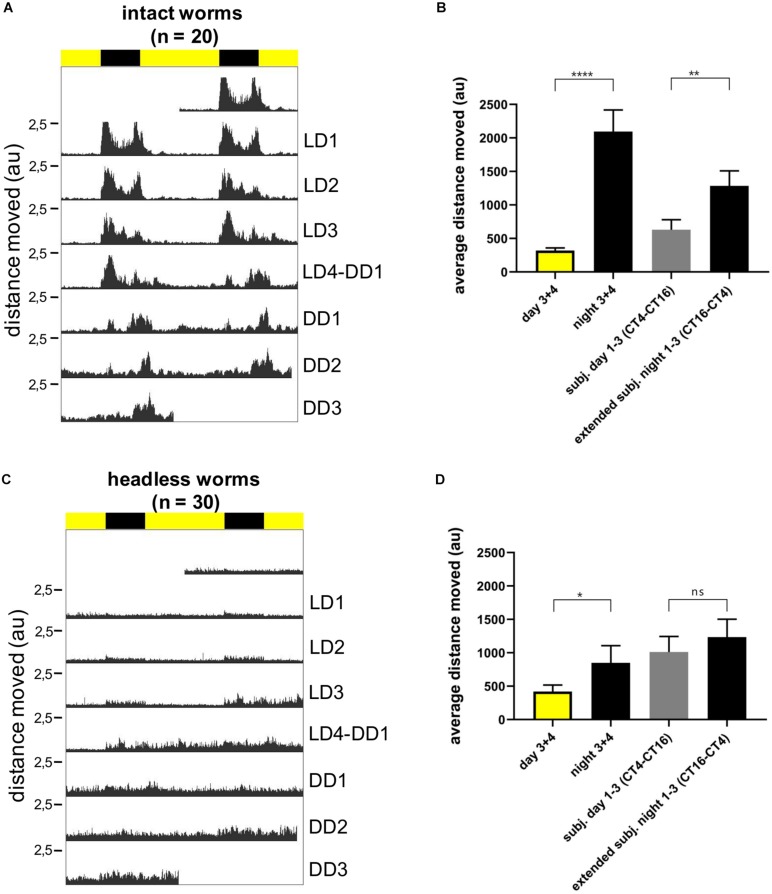
Individual locomotor activity of intact (*n* = 20) and headless (*n* = 30) worms under 16 h light and 8 h dark (LD) conditions followed by 3 days of constant darkness (DD). **(A,C)** Average double plotted actograms of intact **(A)** and headless **(C)** worms. Black bar indicates night hours whereas yellow bar indicates day hours during LD conditions. Decapitation of headless worms was performed at ZT14 1 day before recording (=LD0). **(B,D)** Average distance moved of intact **(B)** and headless worms **(D)** during day and nighttime, as well as during different time periods of constant darkness, i.e., CT4-CT16 for subjective day, and CT16-CT4 for subjective night. The subjective night period was extended to CT4 because under DD conditions activity levels cycle with a ca. 25 h ± 0.22 period (mean ± SEM, see Supplementary Figure 4B), and therefore activity during DD1-3 subjective night moves into the early phase of the subjective day. For day and night time activity, data from LD3 and LD4 were pooled, since during the first 2 days after head removal worms hardly moved and we did not want to bias our results by a potential post-surgery effect. Bars indicate mean ± SEM. *p*-Values were calculated using repeated measures ANOVA followed by Sidak’s multiple comparison test with ^****^p < 0.0001, ^∗∗^*p* < 0.01, and ^*^p < 0.05; au, arbitrary unit.

We next turned to locomotor activity as a read-out for circadian clock activity. We have previously shown that *P. dumerilii* during new moon (circalunar phase of its circalunar clock) exhibits nocturnal circadian locomotor activity, which free-runs under constant darkness for at least 3 days (Zantke et al., 2013, 2014). We meanwhile established an automated worm locomotor behavioral tracking system, which measures worm activity as distance moved of the worm’s center point within 6 min time bins [Ayers et al., 2018, LoopBio (see footnote 1), Vinoth Babu Veedin Rajan *unpublished*]. It should be noted that this new type of analysis measures relative distance moved, compared to the binary (movement: yes or no) manual scoring done by Zantke et al. (2013). Intact worms showed a significant circadian rhythmic locomotor activity under LD and DD conditions (Figures 6A,B, for individual actograms see Supplementary Figure 4). In contrast decapitated worms exhibit an overall severe reduction in movement and rhythmicity (Figures 6C,D, for individual actograms, see Supplementary Figures 5, 6 and Supplementary Movies 1–6. Please note that the maximal values of the *Y*-axis for decapitated animals is 5–10 smaller than for non-decapitated controls in order to visibly display movement). Overall, these data suggest that lack of signals from the head lead to a general suppression of movement and lack of circadian information or synchronization signals for the locomotor output. Headless worms are not generally paralyzed, as they can show spontaneous bursts of movement (Supplementary Figures 5, 6 and Supplementary Movies 1–6) and- depending on their stage at decapitation- can proceed to maturation and the associated behavioral changes (Hauenschild, 1966). Similar to the rhythmic transcript oscillations of the core circadian clock genes in the trunk, acute light functions as a synchronization cue to the periphery of headless worms, but without head or light stimuli a circadian locomotor pattern cannot be maintained by the trunk alone in the majority of the worms.

## Discussion

### Molecular Peripheral Clock

Here, we examine peripheral circadian clock transcript changes and diel rhythms in chromatophore size and locomotor behavior in *P. dumerilii* in the absence and presence of its head. An endogenous circadian rhythm driving body pigmentation change in *P. dumerilii* had been previously proposed based on photographic recordings of isolated groups of body segments during the middle of the day and the night (Röseler, 1969, 1970). With the exception of gills in oysters (Payton et al., 2017), there is no information on fluctuation of circadian clock genes on peripheral tissues or appendages in marine organisms. We document the expression of core circadian clock genes in the periphery of *P. dumerilii*, arguing in favor of a functional circadian peripheral clock and opening an avenue to study the molecular mechanisms of peripheral clocks in marine invertebrates. We show that light/dark cycles can (at least in part) substitute for the head as major synchronizer for continuous peripheral core circadian clock transcript oscillations. We confirm previous work on chromatophore rhythms, which – in contrast to locomotion and transcript oscillations- exhibit free-running rhythms in trunks of headless worms.

As previously reported for the central clock (Zantke et al., 2013), *period* and *bmal* transcripts cycle in antiphase from each other, while *tr-cry* transcripts are neither directly in-phase nor in anti-phase with any of them. Our results on trunks show that cycling of *bmal* and *period* continues under LD conditions, but not under DD independently of the head being present or absent, and can be entrained to an inverted LD cycle on decapitated animals. These results suggest that the peripheral clocks are light dependent (through a yet to be identified set of photoreceptors in the trunk) and either stop, or more likely, desynchronize in the different peripheral cells and tissues (at least as fast as 3 days in DD) in the absence of the head. Consistent with endogenous oscillators desynchronizing over time this desynchronization does not happen immediately upon placement in DD conditions (i.e., DD1). A system with peripheral clocks independent from the central circadian clocks in the brain and entrained directly by environmental signals is reminiscent of *Drosophila melanogaster* (Tomioka et al., 2012). In that sense the peripheral clock in *P. dumerilii* resembles that of insects, and light reception in the trunk likely occurs via Cryptochromes and/or Opsins. Candidates include Go-Opsin1 and rOpsin1 (Backfisch et al., 2013; Ayers et al., 2018).

Light exhibits different effects on the different readouts. In the cases of transcript oscillations and locomotor activity the head is not required for its impact, suggesting that peripheral photoreceptors mediate this signal. Interestingly, different wavelengths appear to have differential peripheral effects on transcript oscillations (red light being able to re-entrain *per* and *bmal* trunk oscillations, but not *tr-cry*), which already indicates the involvement of more than one photoreceptor. It will be interesting for future studies to understand why *tr-cry* transcripts behave differently from *bmal* and *per* transcripts under different light conditions. These differences could be the result of *tr-cry* only being expressed in a subset of tissues/cell types that do not desynchronize as quickly.

As in the case of insects (Tomioka et al., 2012) and mammals (Reppert and Weaver, 2002), questions regarding how peripheral clocks are entrained and the actual mechanisms that peripheral clock use to drive transcriptional changes on various tissues are still questions to be answered in *P. dumerilii*, as is the case for the specific functions of the peripheral clocks.

It should be noted that our experiments were performed on the complete trunk (or at least several segments), which also leaves open questions regarding the peripheral circadian cycling and their synchronizations in specific segmentally repeated organs and tissues.

### Chromatophore Size and the Peripheral Clock

Daily changes in chromatophore size on *P. dumerilii* can be easily used as a visual read out of the circadian clock, adding up to its locomotor activity, circalunar spawning, and clock-related genes as means to study chronobiology in this model system.

Remarkably, the first studies on the cyclic changes of body pigmentation in *P. dumerilii* date back to Hempelmann (1939). Based on this and further classical work, these changes in body pigmentation were already attributed to an endogenous circadian rhythm present in each segment of the worm’s body (Fischer, 1965; Röseler, 1969, 1970), pointing at the existence of peripheral circadian clocks long before their molecular mechanism had been unraveled and circadian peripheral oscillations were proven to exist in the peripheral tissues in mammals (Tosini and Menaker, 1996; Balsalobre et al., 1998; Schibler et al., 2003; Dibner et al., 2010). Our analyses support the classical studies on Platynereis, in that chromatophore size in the body of *P. dumerilii* is higher during day time, with a major drop before the night begins; which argues in favor of a clock-driven manifestation and not a direct light response. The average magnitude of this change corresponds with previous quantitative reports by Fischer (1965). We report that individuals placed in DD for 5 days still exhibit a significant daily difference on chromatophore size, although its maximum value decreases by 25% compared to the initial LD conditions. It has been reported that such cycling starts to fade on individual chromatophores after 7 days in DD (Fischer, 1965), but we did not test for the long term stability of the cycle for individual chromatophores. Noticeably, a similar circadian cycling of chromatophore size on DD and inverted LD cycles has been shown for the marine isopod *E. pulchra* (Zhang et al., 2013), posing the questions if this regulation is similar.

Changes in chromatophore size with a circadian cycling is commonly seen in crustaceans (Fingerman, 1955; Fingerman and Yamamoto, 1967; Fingerman et al., 1969; Zhang et al., 2013). There is usually an increase in size during the day thought to be related to UV protection (Darnell, 2012), but an inverted pattern of bigger chromatophore size during the night, to possibly enable individuals to camouflage with the substrate, can also be found (Stevens et al., 2013). The fact that *P. dumerilii* is mostly active during the night (Zantke et al., 2013), when pigmentation is lower, does not argue in favor of a camouflage role; especially since pigmentation does not respond to changes in background brightness (Fischer, 1965). The most parsimonious option is therefore, a role of pigmentation in protecting the animal from UV light. It should be noted, however, that it has also been often proposed, but not tested, that circadian changes in pigmentation might be a mechanism related to energy saving (Stevens, 2016).

Remarkably, in our hands the re-entrainment of chromatophore rhythms by light requires the presence of the head. This might be either because the required photoreceptor(s) are located in the head or because hormonal feedback signals, such as *Pdu*-PDF (pigment dispersing factor) (Shahidi et al., 2015), are required for the synchronization process. It is likely that pigmentation in *P. dumerilii* is controlled by a hormonal process, as in some crustaceans (Fingerman and Yamamoto, 1967; Fingerman et al., 1969; Nery et al., 1999), which in turn is governed by the central clock. The hormonal nature of the cycling on chromatophore size has been further supported by the immediate reaction of chromatophores, present on isolated skin tissue, when coelomic fluid from *P. dumerilii* during a given time point (e.g., day or night) is added (Röseler, 1970).

Finally, while we overall confirm previous work on the chromatophore rhythms in the trunk of *P. dumerilii*, there is one clear difference between our findings and that of Röseler (1970). Her work shows that chromatophore rhythms can still be re-entrained by white light even in the absence of the prostomium (head), whereas in our hands decapitated animals do not re-entrain their chromatophore rhythm in response to white light. We identified two main reasons that might explain this discrepancy. The materials and methods of her paper do not state the light intensity and spectrum. It is thus possible that this strongly deviates from our conditions. The other difference is the extent of head removal. In our study we removed the head including the jaw piece, whereas Röseler (1970) specifies prostomium-removal, which implies that her worms still possessed the jaw and the surrounding tissue. This region possesses multiple neurons and neurosecretory cells, which could be important for proper re-entrainment. Further work will certainly help to disentangle these differences.

## Conclusion

We find that the overall circadian clock transcript oscillations of the trunk are under strong control of the LD cycle and do not show synchronized oscillation under constant darkness, irrespective of the absence or presence of heads. In the absence of heads, locomotor activity is also strongly coordinated by the LD cycle and significantly reduced. In contrast, circadian changes of body pigmentation in the trunk free-run over several days in constant darkness, even in the absence of the head. Jointly these data indicate that autonomous peripheral clocks exist in the trunk of the bristle worm, coordinating for instance pigmentation. However, the synchronization of rhythmic circadian oscillations in other peripheral tissues and their respective output are more strongly coordinated by light than by the circadian oscillator positioned in the head of the worm. Our data build a basis for future analyses of the multiple clocks of the bristle worm, but also suggest that peripheral clocks should be taken into consideration when studying other organisms with circadian and non-circadian oscillators.

## Data Availability

All datasets generated for this study are included in the manuscript and/or the Supplementary Files.

## Ethics Statement

All animal work was conducted according to the Austrian and European guidelines for animal research. Please note that our work is performed on invertebrates, which according to these guidelines do not require special animal experimental permissions or committees.

## Author Contributions

EA and KT-R designed the study and wrote the manuscript. MZ reviewed and commented on the manuscript, and contributed to locomotor activity experiments and measurement of light spectra and intensities. MW and MZ performed the qPCRs for headless worms DD1. EA performed all other experiments. EA, MW, KT-R, and MZ did the data analysis, interpretation, and discussion.

## Conflict of Interest Statement

The authors declare that the research was conducted in the absence of any commercial or financial relationships that could be construed as a potential conflict of interest.

## Footnotes

1 www.loopbio.com
