# Supplementary material for: Differential Impacts of the Head on Platynereis dumerilii Peripheral Circadian Rhythms
Source: Front Physiol. 2019 Jul 11;10:900. doi: 10.3389/fphys.2019.00900 (PMC6638195; doi:10.3389/fphys.2019.00900)
Supplement: Supplementary file 1 [file Presentation_1.pdf]

Supplementary Figure 1. Graphical view of spectra and intensity of light used during the experiments.

Supplementary Figure 2. qPCR results of additional clock-related genes in trunks and of *bmal*, *period* and *tr-cry* on different segments of the trunk.

Supplementary Figure 3. Changes in chromatophores size on Individual replicas of animals used on Figure 5D.

Supplementary Figure 4. Individual behavioral actograms of intact animals used on Figure 6.

Supplementary Figure 5. Individual behavioral actograms of decapitated animals used on Figure 6.

Supplementary Figure 6. Actograms of exemplary headless worms were the time is indicated at which supplementary movie sections 1-6 were recorded.

Supplementary Figure legends: Description of the Supplementary Figure contents.

Supplementary Data 1. Detailed statistical results of two-way ANOVAs and related post-hoc analyses used on Figures 1, 2, 3 and 5.

Supplementary Data 2._Individual activity data of intact and headless worms. These are the primary data used for Fig. 6 and Supplementary Fig. 4, 5 and 6

Supplementary Movies 1-6. Exemplary movies of active and inactive headless worms recorded during day and night. See Supplementary Fig. 6 for details on the time of recording and tracked activity trajectories over the whole course of the experiment.

(A)

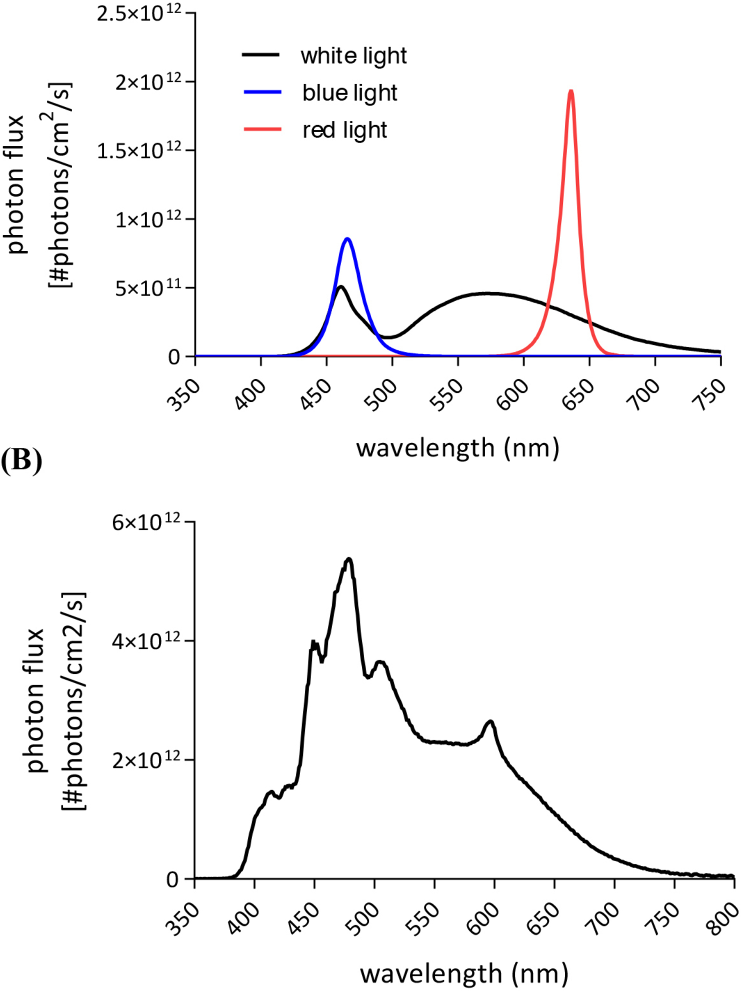

Supplementary Figure 1.

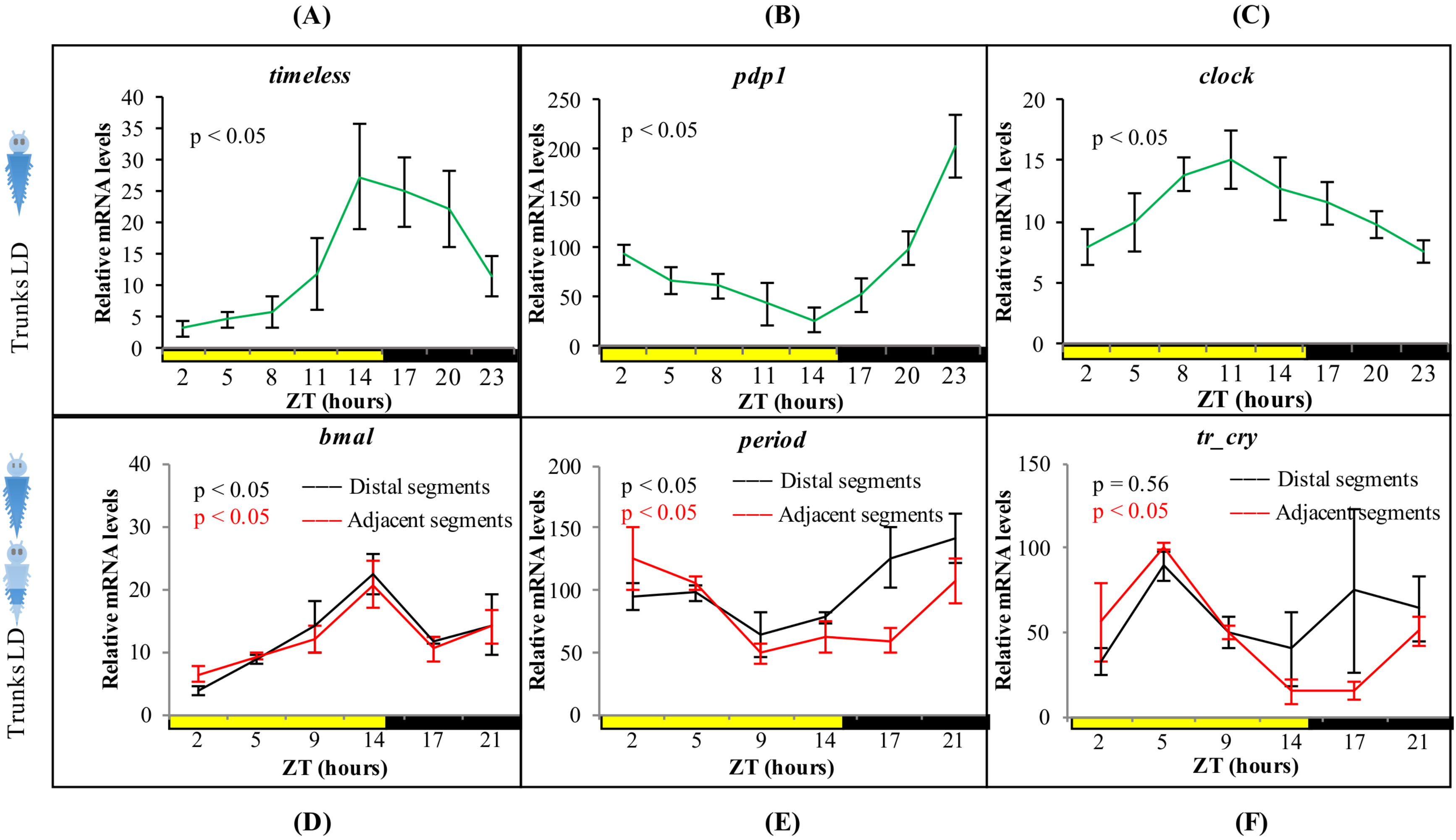

Supplementary Figure 2..

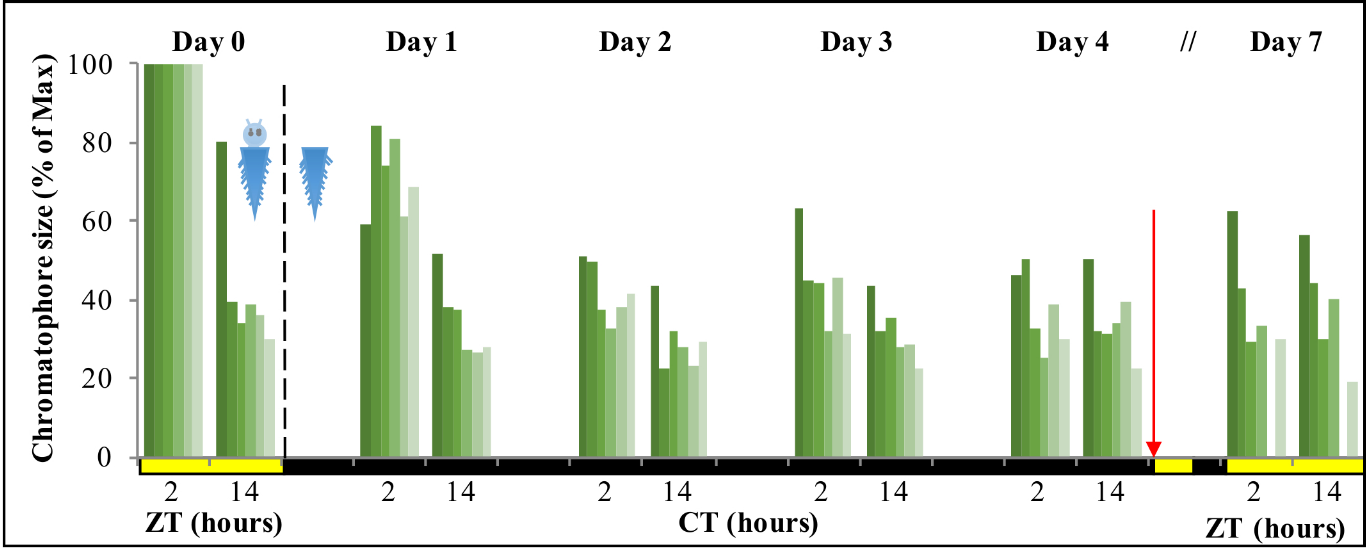

Supplementary Figure 3.

(A)

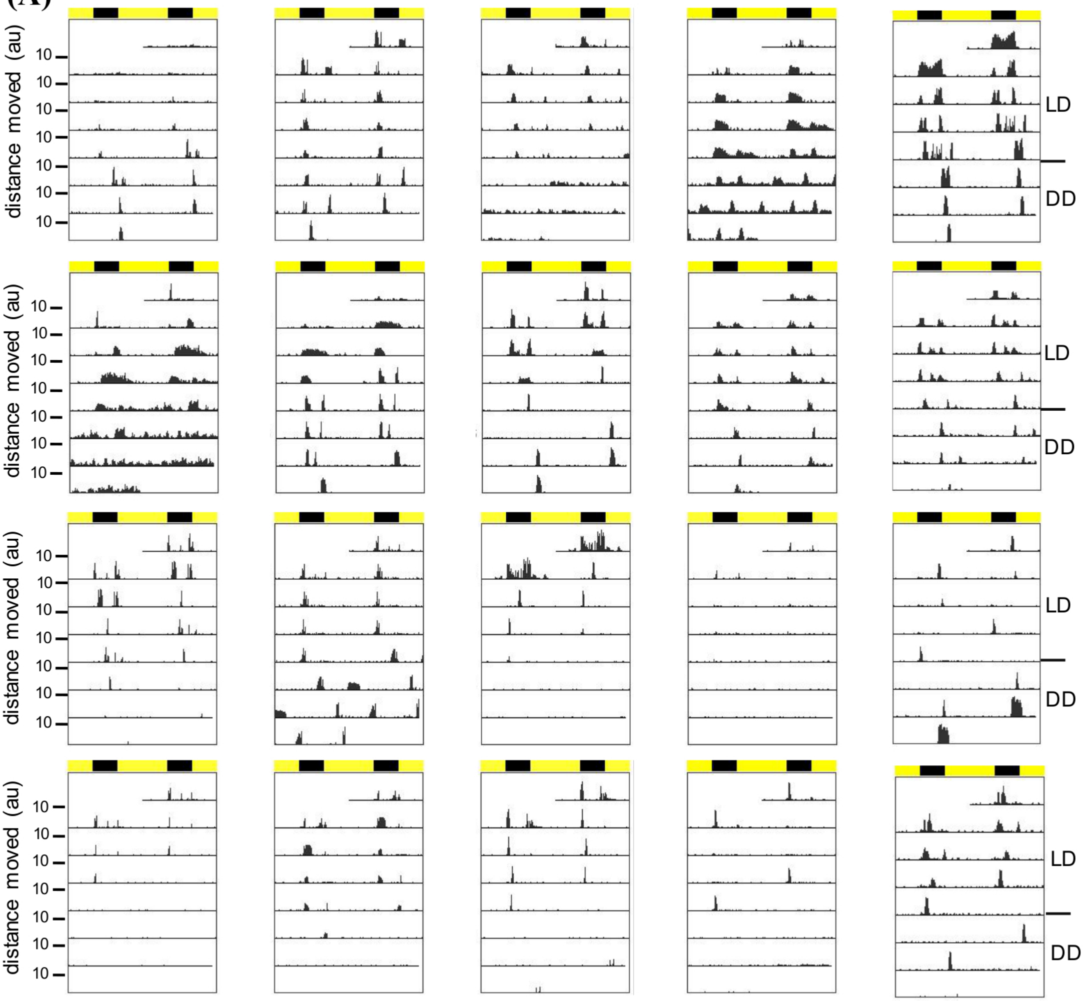

(B)

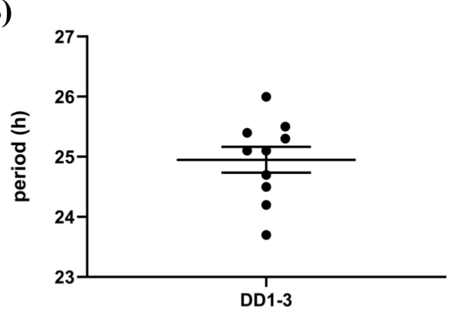

Supplementary Figure 4

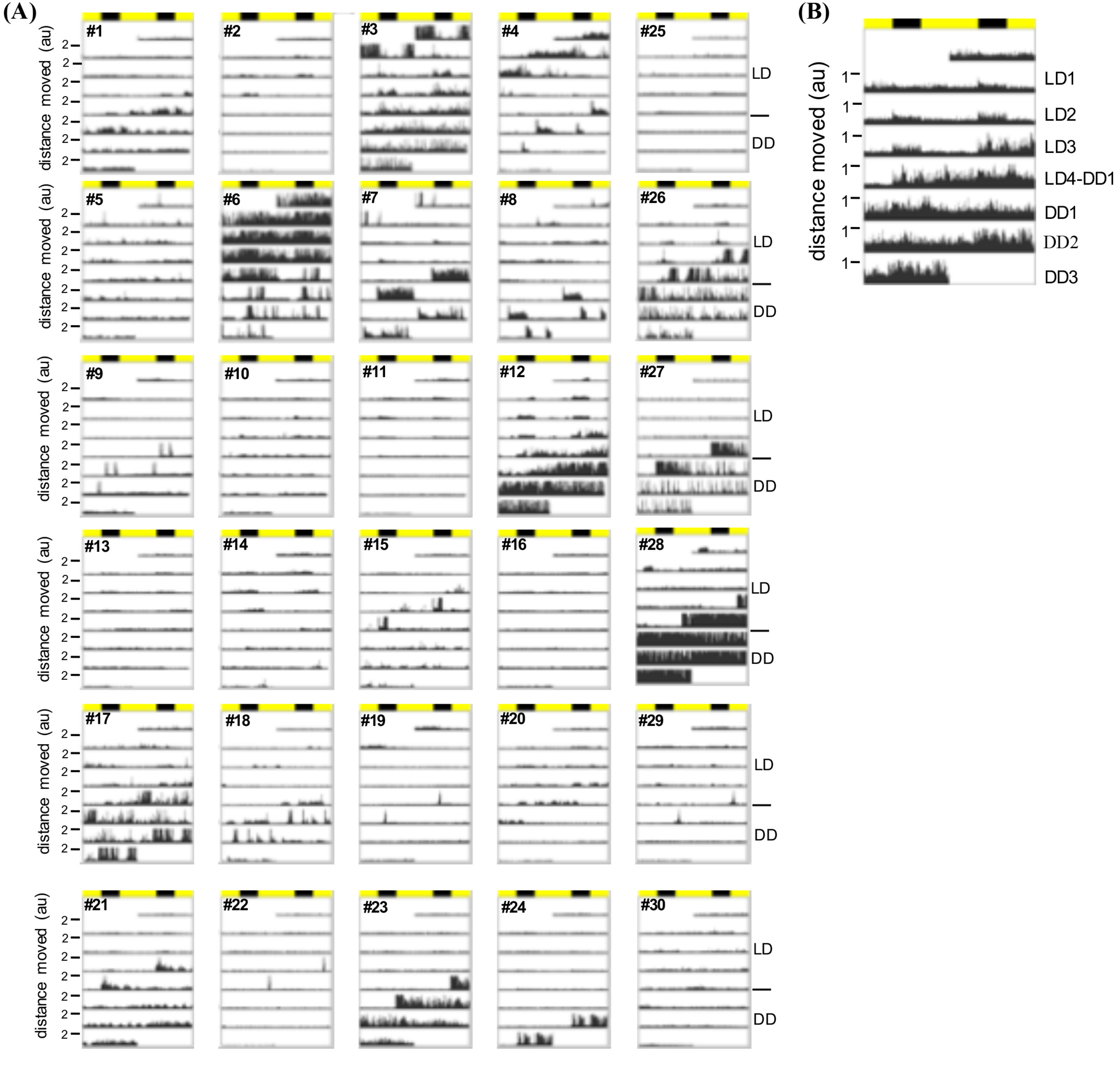

Supplementary Figure 5

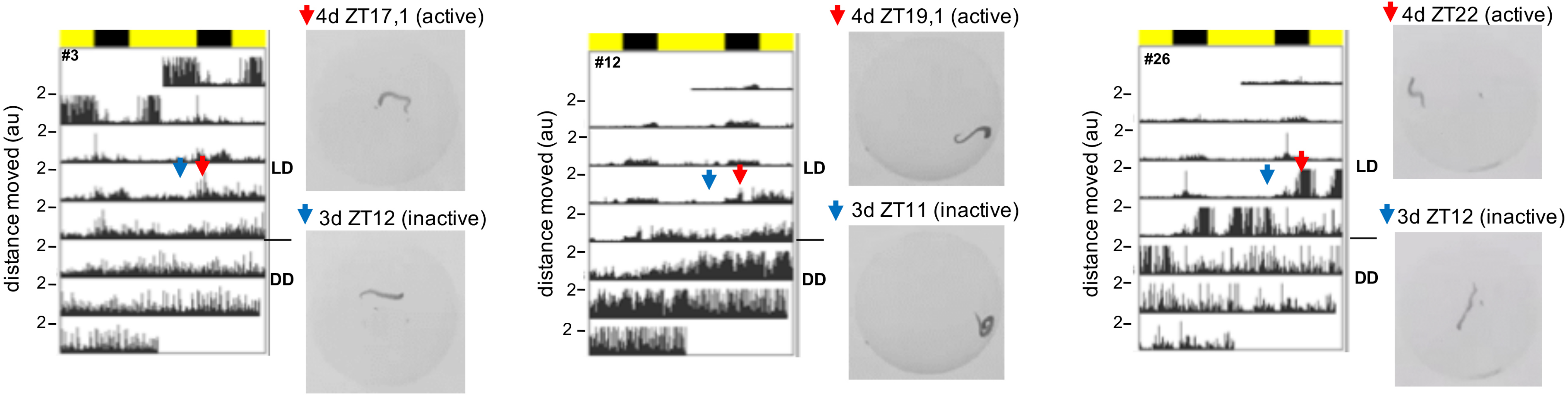

Supplementary Figure 6

Supplementary Figure legends:

Supplementary Figure 1. (A) Spectra and intensity of light used for molecular and chromatophore assays. (B) White light spectrum used in locomotor activity assays.

Supplementary Figure 2. Relative transcript levels of (A) *timeless*, (B) *pdp1* and (C) *clock* in trunks of intact animals (i.e. not decapitated) under standard LD conditions (n= 5 to 8 per ZT point); and of (D) bmal, (E) period and (F) tr-cry on the last 5-7 segments of the body (black line) or the adjacent 5-7 segments towards the anterior part of the animal (red line) (n=3 in all cases). ZT= Zeitgeber Time. p-value estimated on a single factor ANOVA (alpha= 0.05). Error bars denote SEM

Supplementary Figure 3. Individual replicas of animals used on Figure 5D, showing chromatophore size at ZT/CT 12 and ZT/CT 14 over seven consecutive days. Dashed line indicates decapitation and placement in DD conditions. Red arrow indicates re-placement under LD conditions (n=6). ZT= Zeitgeber Time and CT= Circadian Time.

Supplementary Figure 4. (A) Individual locomotor activity of intact worms under 16h light and 8h dark (LD) conditions followed by three days of constant darkness (DD) depicted in individual double plotted actograms. Black bar indicates night hours whereas yellow bar indicates day hours during LD conditions. Decapitation of headless worms was performed at ZT14 one day before recording (=LD0). (B) Lomb-Scargle periodogram analysis of activity data recorded under DD1-3 reveals a period length of 25.0h ± 0.22 h (n=10, mean ± SEM). Only periods that had a power value >20 were considered (power values of >10 were already classified to be significantly rhythmic (p<0.05) by the Lomb-Scargle analyses, but we used a more stringent cut-off to reduce the probability of detecting false-positive periods). Out of the 20 worms tested only the first 10 worms depicted in (A) show power values >20, and therefore only these were considered to calculate the mean period length. au=arbitrary unit

Supplementary Figure 5. (A) Individual locomotor activity of headless worms under 16h light and 8h dark (LD) conditions followed by three days of constant darkness (DD) depicted in individual double plotted actograms. Black bar indicates night hours whereas yellow bar indicates day hours during LD conditions. Decapitation of headless worms was performed at ZT14 one day before recording (=LD0). (B) Average double plotted actogram of all 30 worms depicted in (A). Note that maximum value of y-axis in (A) is five times and in (B) ten times smaller than in Fig S4, in order to make also subtle locomotor activity visible. au=arbitrary unit

Supplementary Figure 6. Exemplary movies of headless worm recorded during light or dark conditions. Double plotted actogrammes on the left show activity trajectories of an individual worm over the whole course of the experiment, while red and blue arrows indicate the time when the depicted movie was recorded. Movies are attached in the supplement (Supplementary movie 1-6). Note that movies are accelerated 1,7 times. au=arbitrary unit
