# Supplementary material for: Differential Impacts of the Head on Platynereis dumerilii Peripheral Circadian Rhythms
Source: Front Physiol. 2019 Jul 11;10:900. doi: 10.3389/fphys.2019.00900 (PMC6638195; doi:10.3389/fphys.2019.00900)
Supplement: Supplementary file 2 [file Data_Sheet_1.pdf]

Additional File 1
Detailed statistic results for two way ANOVA and post-hoc analyses for figures 1, 2 and 3. For more details, see materials and methods.

<u>Figure 1:</u>
1) To evaluate if overall relative mRNA levels levels are comparable over treatments:

1.1) for bmal:
> pairs(marginal, adjust="tukey")

contrast	estimate	SE	df	t.ratio	p.value
HeadsLD - TailsDD	21.8	2.26	155	9.629	<.0001
HeadsLD - TailsLD	20.6	2.20	155	9.362	<.0001
TailsDD - TailsLD	-1.2	1.38	155 -	0.866	0.6626

Results are averaged over the levels of: ZT
P value adjustment: tukey method for comparing a family of 3 estimates

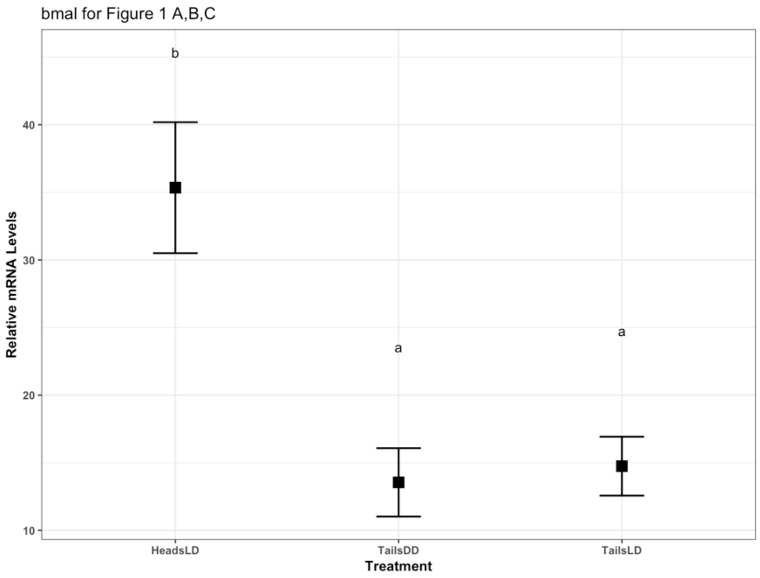

Treatment	lsmean	SE	df l	ower.CL	upper.CL	.group
TailsDD	13.5	1.048	155	11.0	16.1	a
TailsLD	14.7	0.903	155	12.6	16.9	a
HeadsLD	35.3	2.007	155	30.5	40.2	b

Results are averaged over the levels of: ZT
Confidence level used: 0.95
Conf-level adjustment: sidak method for 3 estimates
P value adjustment: tukey method for comparing a family of 3 estimates
significance level used: alpha = 0.05

1.2) for period:

> pairs(marginal, adjust="tukey")

contrast	estimate	SE	df	t.ratio	p.value
HeadsLD - TailsDD	99.8	10.75	155	9.287	<.0001
HeadsLD - TailsLD	72.7	10.45	155	6.958	<.0001
TailsDD - TailsLD	-27.1	6.57	155	-4.131	0.0002

Results are averaged over the levels of: ZT
P value adjustment: tukey method for comparing a family of 3 estimates

Treatment	lsmean	SE	df	lower.CL	upper.CL .	group
TailsDD	74	4.98	155	62.0	86	a
TailsLD	101	4.29	155	90.8	112	b
HeadsLD	174	9.53	155	150.8	197	c

Results are averaged over the levels of: ZT
Confidence level used: 0.95
Conf-level adjustment: sidak method for 3 estimates
P value adjustment: tukey method for comparing a family of 3 estimates
significance level used: alpha = 0.05

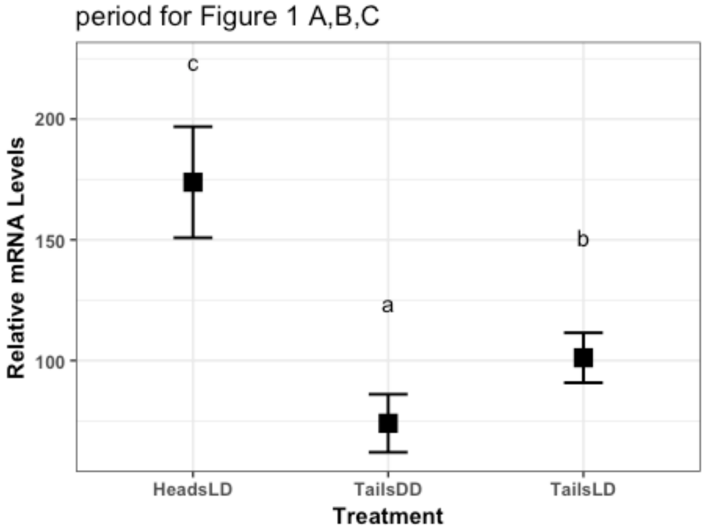

1.3) for tr-cry

> pairs(marginal, adjust="tukey")

contrast	estimate	SE	df	t.ratio	p.value
HeadsLD - TailsDD	58.38943	7.76	155	7.520	<.0001
HeadsLD - TailsLD	58.37969	7.55	155	7.736	<.0001
TailsDD - TailsLD	-0.00974	4.74	155 -	0.002	1.0000

Results are averaged over the levels of: ZT

P value adjustment: tukey method for comparing a family of 3 estimates

Treatment lsmean	SE	df	lower.CL	upper.CL	group
TailsDD	43.9 3.59	155	35.2	52.5	a
TailsLD	43.9 3.10	155	36.4	51.3	a
HeadsLD	102.3 6.88	155	85.6	118.9	b

Results are averaged over the levels of: ZT

Confidence level used: 0.95

Conf-level adjustment: sidak method for 3 estimates

P value adjustment: tukey method for comparing a family of 3 estimates

significance level used: alpha = 0.05

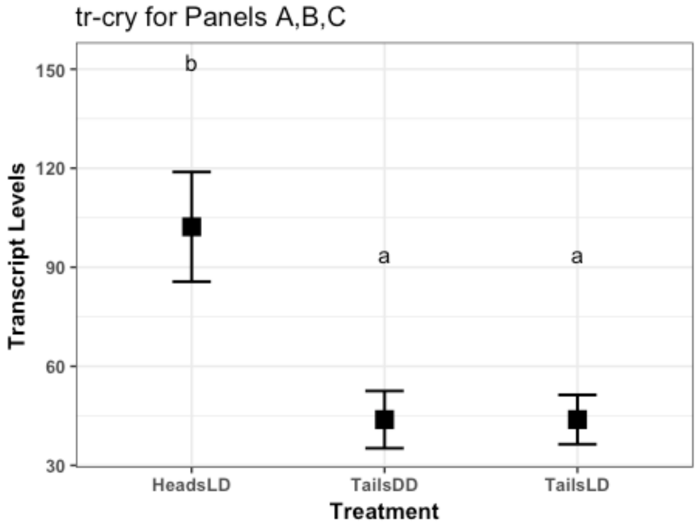

2) Evaluation of data set for ANOVA assumptions (Homogeneity of variances and Normality by Histogram of Residuals). This includes only panles A,B (i.e. Heads LD and Trunks LD) for tr-cry

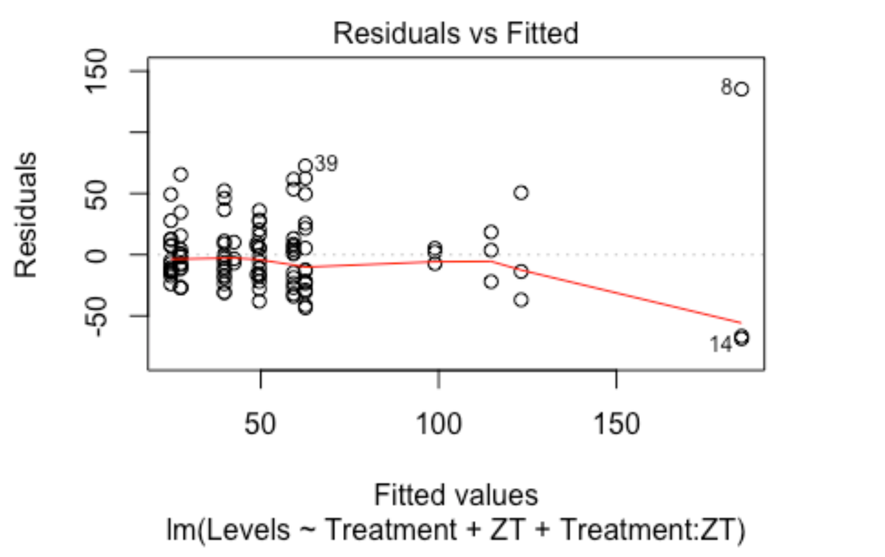

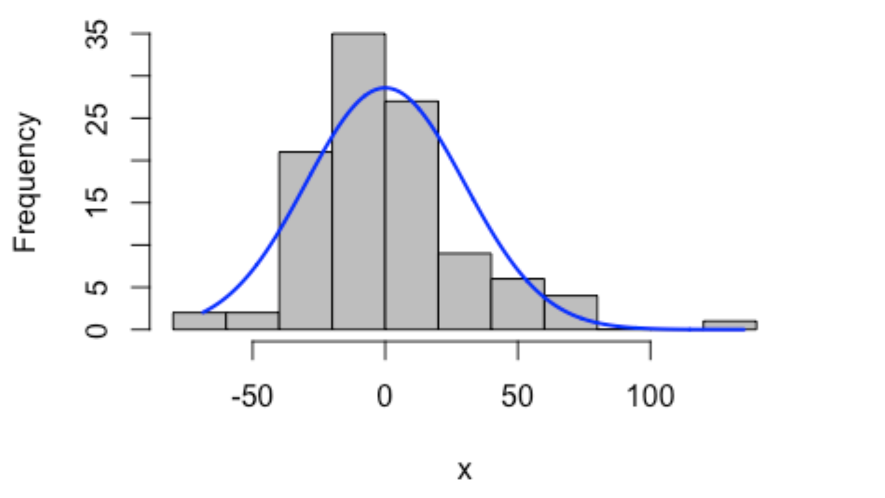

2.1) Anova Table (Type III tests)

Response: Levels

	Sum Sq	Df	F value	Pr(>F)
(Intercept)	319644	1	320.9461	< 2.2e-16 ***
Treatment	51022	1	51.2294	1.716e-10 ***
ZT	48996	5	9.8392	1.334e-07 ***
Treatment:ZT	26809	5	5.3837	0.000211 ***
Residuals	94615 95			

--- Signif. codes: 0 ‘***’ 0.001 ‘**’ 0.01 ‘*’ 0.05 ‘.’ 0.1 ‘ ’ 1

2.2) Post hoc pairwise (marginal) Tukey analysis for the interaction.

Grouped by ZT

	contrast	Treatment	estimate	SE	df	t.ratio	p.value
**1**	ZT14 - ZT17	HeadsLD	-6.468123	25.76746	95	-0.2510191	9.99860E-01
**2**	ZT14 - ZT2	HeadsLD	-72.274959	25.76746	95	-2.8048927	6.53413E-02
**3**	ZT14 - ZT21	HeadsLD	-56.473206	25.76746	95	-2.1916482	2.51376E-01
**4**	ZT14 - ZT5	HeadsLD	-142.699513	25.76746	95	-5.5379737	3.99531E-06
**5**	ZT14 - ZT9	HeadsLD	-80.722576	25.76746	95	-3.1327332	2.70987E-02
**6**	ZT17 - ZT2	HeadsLD	-65.806836	25.76746	95	-2.5538736	1.19251E-01
**7**	ZT17 - ZT21	HeadsLD	-50.005083	25.76746	95	-1.9406291	3.84315E-01
**8**	ZT17 - ZT5	HeadsLD	-136.23139	25.76746	95	-5.2869546	1.15918E-05
**9**	ZT17 - ZT9	HeadsLD	-74.254453	25.76746	95	-2.8817142	5.36579E-02
**10**	ZT2 - ZT21	HeadsLD	15.801753	25.76746	95	0.6132445	9.89831E-01
**11**	ZT2 - ZT5	HeadsLD	-70.424554	25.76746	95	-2.733081	7.81338E-02
**12**	ZT2 - ZT9	HeadsLD	-8.447617	25.76746	95	-0.3278405	9.99482E-01
**13**	ZT21 - ZT5	HeadsLD	-86.226307	25.76746	95	-3.3463255	1.44901E-02
**14**	ZT21 - ZT9	HeadsLD	-24.24937	25.76746	95	-0.941085	9.34725E-01
**15**	ZT5 - ZT9	HeadsLD	61.976937	25.76746	95	2.4052405	1.64917E-01
**16**	ZT14 - ZT17	TailsLD	2.754897	11.52356	95	0.2390666	9.99890E-01
**17**	ZT14 - ZT2	TailsLD	-31.723879	11.72753	95	-2.7050775	8.36563E-02
**18**	ZT14 - ZT21	TailsLD	-22.109222	11.52356	95	-1.9186107	3.97384E-01
**19**	ZT14 - ZT5	TailsLD	-12.25159	11.52356	95	-1.0631777	8.94632E-01
**20**	ZT14 - ZT9	TailsLD	-35.059103	11.52356	95	-3.0423852	3.48878E-02
**21**	ZT17 - ZT2	TailsLD	-34.478777	11.72753	95	-2.939986	4.60324E-02
**22**	ZT17 - ZT21	TailsLD	-24.864119	11.52356	95	-2.1576772	2.67461E-01
**23**	ZT17 - ZT5	TailsLD	-15.006487	11.52356	95	-1.3022442	7.83119E-01
**24**	ZT17 - ZT9	TailsLD	-37.814	11.52356	95	-3.2814517	1.75975E-02
**25**	ZT2 - ZT21	TailsLD	9.614657	11.72753	95	0.8198365	9.63194E-01
**26**	ZT2 - ZT5	TailsLD	19.47229	11.72753	95	1.6603913	5.61112E-01
**27**	ZT2 - ZT9	TailsLD	-3.335223	11.72753	95	-0.2843926	9.99742E-01
**28**	ZT21 - ZT5	TailsLD	9.857632	11.52356	95	0.855433	9.55949E-01
**29**	ZT21 - ZT9	TailsLD	-12.949881	11.52356	95	-1.1237745	8.70354E-01
**30**	ZT5 - ZT9	TailsLD	-22.807513	11.52356	95	-1.9792075	3.61903E-01

Blue bars correspond to confidence intervals (95%) for the EMMs (Estimated Marginal Means). Red arrows indicate the comparisons among them. If an arrow from one mean overlaps an arrow from another group, the difference is not significant.

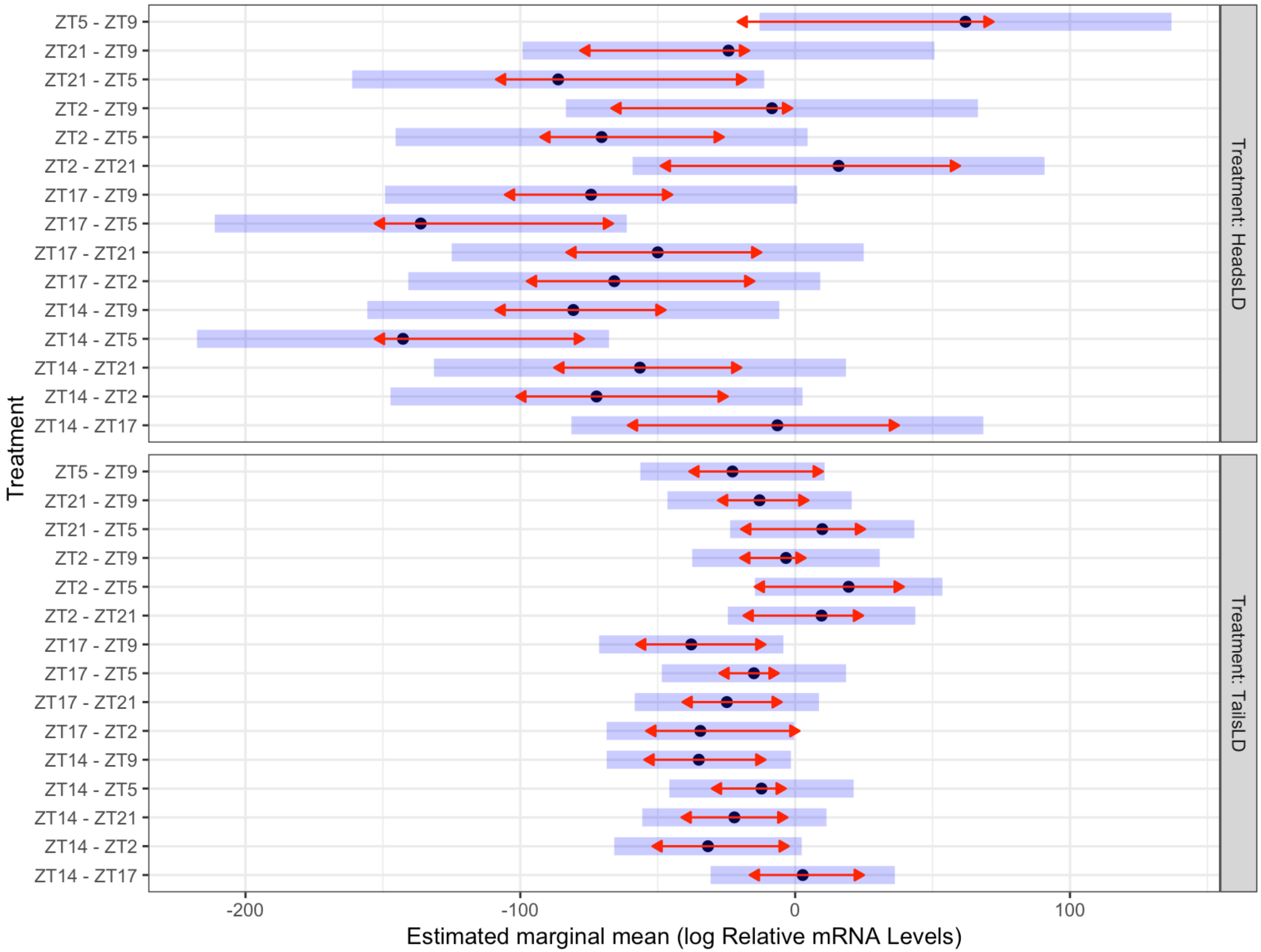

Grouped by treatment

	Contrast	ZT	Estimate	SE	Df	t.ratio	p.value
**1**	HeadsLD – TailsLD	ZT14	15.00478	19.95939	95	0.7517654	4.540506e-01
**2**	HeadsLD – TailsLD	ZT17	24.22780	19.95939	95	1.2138547	2.278123e-01
**3**	HeadsLD – TailsLD	ZT2	55.55586	20.07784	95	2.7670232	6.799741e-03
**4**	HeadsLD – TailsLD	ZT21	49.36876	19.95939	95	2.4734606	1.515709e-02
**5**	HeadsLD – TailsLD	ZT5	145.45270	19.95939	95	7.2874328	9.258643e-11
**6**	HeadsLD - TailsLD	ZT9	60.66825	19.95939	95	3.0395847	3.060505e-03

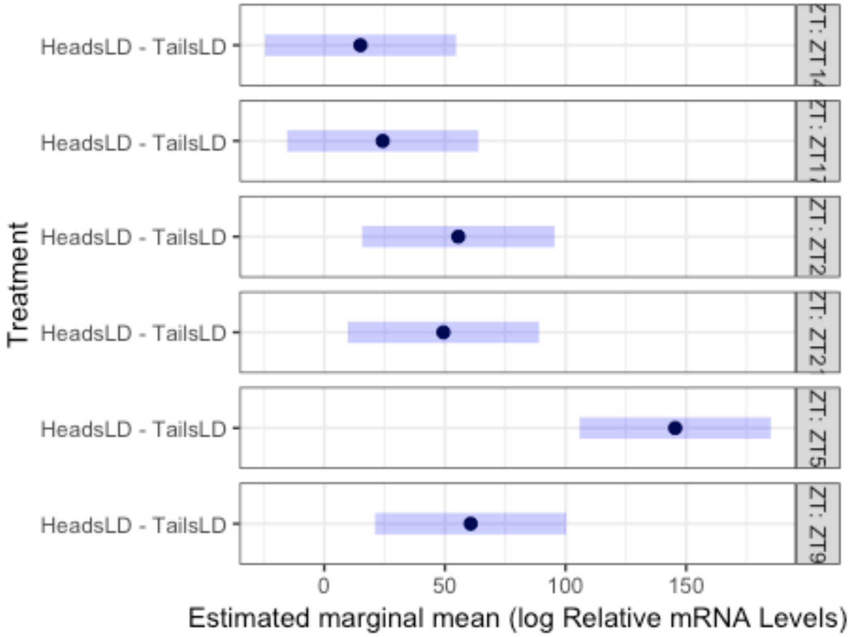

Figure 2

1) To evaluate if overall relative mRNA levels levels are comparable over treatments:

1.1) for bmal:

> pairs(marginal, adjust="tukey")

contrast	estimate	SE	df	t.ratio	p.value
TailsDD1 - TailsDD3	-0.943	1.18	88	-0.801	0.7032
TailsDD1 - TailsLD	-7.135	1.20	88	-5.941	<.0001
TailsDD3 - TailsLD	-6.192	1.20	88 -	5.156	<.0001

Results are averaged over the levels of: ZT

Treatment	lsmean	SE	df	lower.CL	upper.CL	.group
TailsDD1	11.6	0.832	88	9.62	13.7	a
TailsDD3	12.6	0.832	88	10.56	14.6	a
TailsLD	18.8	0.866	88	16.67	20.9	b

Results are averaged over the levels of: ZT

Confidence level used: 0.95

Conf-level adjustment: sidak method for 2 estimates

significance level used: alpha = 0.05

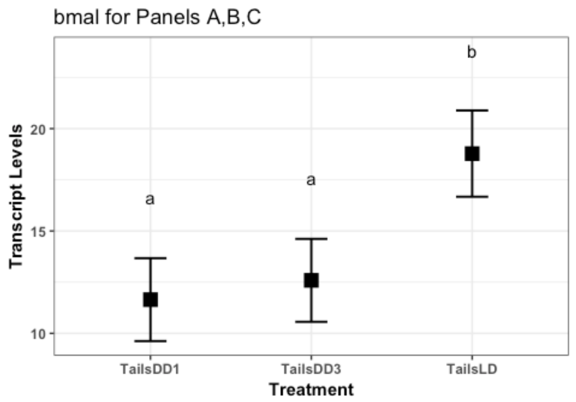

1.2) for per:

> pairs(marginal, adjust="tukey")

contrast	estimate	SE	df	t.ratio	p.value
TailsDD1 - TailsDD3	117.5	6.82	88	17.227	<.0001
TailsDD1 - TailsLD	103.6	6.96	88	14.885	<.0001
TailsDD3 - TailsLD	-13.9	6.96	88	-1.994	0.1197

Results are averaged over the levels of: ZT

Treatment	lsmean	SE	df	lower.CL	upper.CL .	group
TailsDD3	61.4	4.82	88	49.7	73.2	a
TailsLD	75.3	5.02	88	63.1	87.5	a
TailsDD1	178.9	4.82	88	167.2	190.7	b

Results are averaged over the levels of: ZT

Confidence level used: 0.95

Conf-level adjustment: sidak method for 2 estimates

significance level used: alpha = 0.05

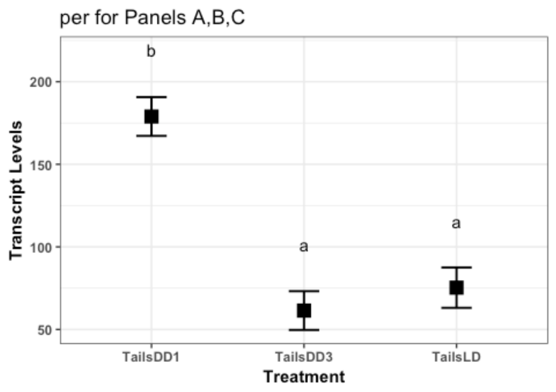

1.3) for tr-cry:

> pairs(marginal, adjust="tukey")

contrast	estimate	SE	df	t.ratio	p.value
TailsDD1 - TailsDD3	75.0	5.92	88	12.660	<.0001
TailsDD1 - TailsLD	44.8	5.97	88	7.495	<.0001
TailsDD3 - TailsLD	-30.2	5.92	88	-5.103	<.0001

Results are averaged over the levels of: ZT

Treatment	lsmean	SE	df	lower.CL	upper.CL	.group
TailsDD3	46.0	4.16	88	35.9	56.1	a
TailsLD	76.2	4.22	88	66.0	86.5	b
TailsDD1	121.0	4.22	88	110.7	131.3	c

Results are averaged over the levels of: ZT

Confidence level used: 0.95

Conf-level adjustment: sidak method for 2 estimates

significance level used: alpha = 0.05

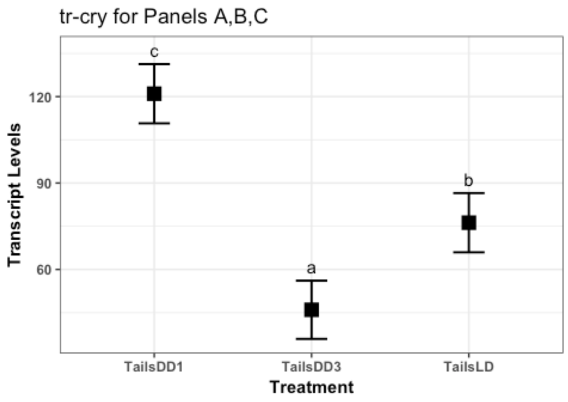

Figure 3:

1) bmal

Evaluation of data set for ANOVA assumptions (Homogeneity of variances and Normality by Histogram of Residuals).

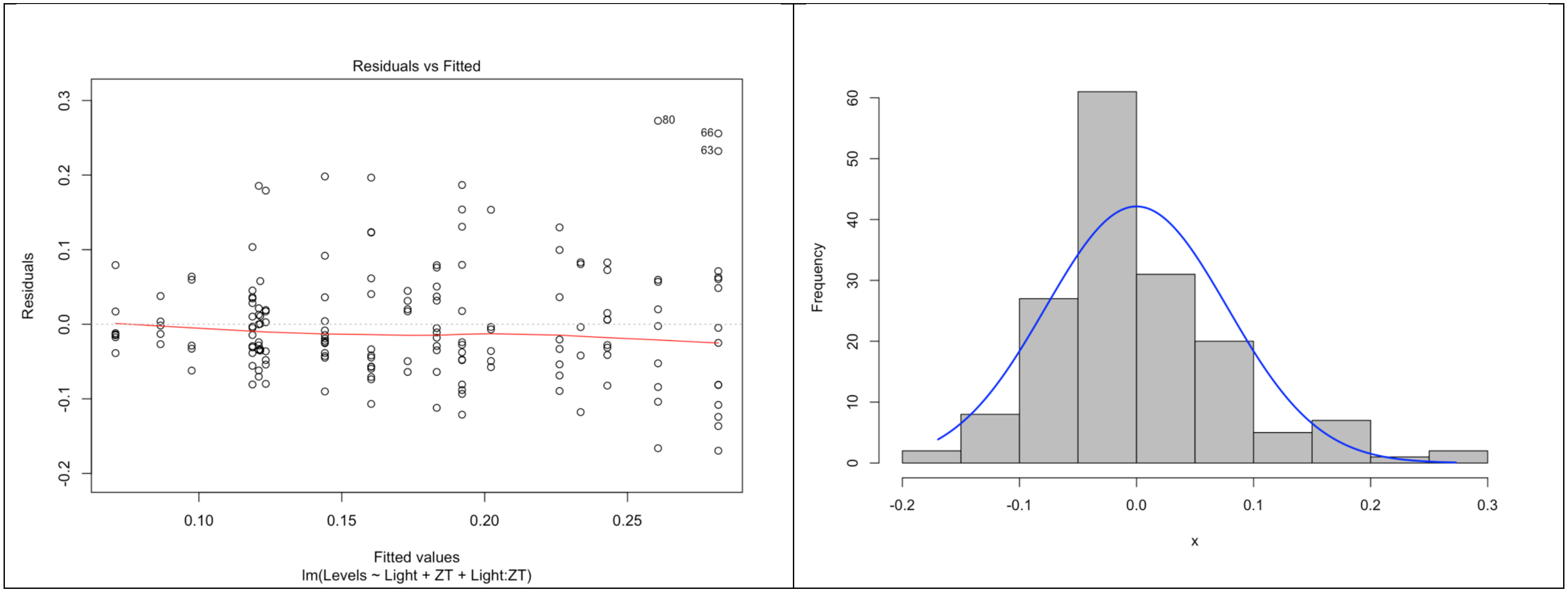

1.1)Two-Way ANOVA (Type III)

	Sum Sq	Df	F value	Pr(>F)
**(Intercept)**	4.01685949	1	597.215661	1.871421e-53
**Light**	0.01760748	2	1.308916	2.732632e-01
**ZT**	0.42678855	5	12.690750	3.039972e-10***
**Light:ZT**	0.09389924	10	1.396068	1.874610e-01
**Residuals**	0.98199281	146	NA	N

Signif. codes: 0 ‘***’ 0.001 ‘**’ 0.01 ‘*’ 0.05 ‘.’ 0.1 ‘ ’ 1

1.2) Post hoc pairwise (marginal) Tukey analysis for ZT effect.

	contrast	estimate	SE	df	t.ratio	p.value
**1**	ZT14 - ZT17	0.02589578	0.02307217	146	1.1223817	8.714074e-01
**2**	ZT14 - ZT2	0.11192563	0.02388684	146	4.6856605	9.162104e-05
**3**	ZT14 - ZT21	0.07853065	0.02357442	146	3.3311810	1.371388e-02
**4**	ZT14 - ZT5	0.09121058	0.02360585	146	3.8638977	2.274888e-03
**5**	ZT14 - ZT9	-0.04626762	0.02338496	146	-1.9785201	3.596757e-01
**6**	ZT17 - ZT2	0.08602985	0.02410313	146	3.5692400	6.335750e-03
**7**	ZT17 - ZT21	0.05263487	0.02379355	146	2.2121492	2.385392e-01
**8**	ZT17 - ZT5	0.06531480	0.02382469	146	2.7414758	7.332839e-02
**9**	ZT17 - ZT9	-0.07216340	0.02360585	146	-3.0570136	3.124204e-02
**10**	ZT2 - ZT21	-0.03339498	0.02458433	146	-1.3583850	7.516262e-01
**11**	ZT2 - ZT5	-0.02071505	0.02461446	146	-0.8415806	9.591706e-01
**12**	ZT2 - ZT9	-0.15819325	0.02440271	146	-6.4826099	1.946959e-08
**13**	ZT21 - ZT5	0.01267992	0.02431139	146	0.5215630	9.952505e-01
**14**	ZT21 - ZT9	-0.12479827	0.02409698	146	-5.1790013	1.070119e-05
**15**	ZT5 - ZT9	-0.13747819	0.02412772	146	-5.6979347	9.606779e-07

Results are averaged over the levels of: Light
P value adjustment: tukey method for comparing a family of 6 estimates

1.3) Pairwise comparisons of Estimated Marginal Means (CLD)

ZT	lsmean	SE	df	lower.CL	upper.CL	.group
ZT2	0.1004866	0.01759570	146	0.05355558	0.1474177	a
ZT5	0.1212017	0.01721230	146	0.07529322	0.1671101	ab
ZT21	0.1338816	0.01716917	146	0.08808818	0.1796750	ab
ZT17	0.1865165	0.01647277	146	0.14258048	0.2304525	bc
ZT14	0.2124123	0.01615465	146	0.16932476	0.2554998	cd
ZT9	0.2586799	0.01690810	146	0.21358279	0.3037770	d

Results are averaged over the levels of: Light
Confidence level used: 0.95. significance level used: alpha = 0.05
Conf-level adjustment: sidak method for 6 estimates
P value adjustment: tukey method for comparing a family of 6 estimates

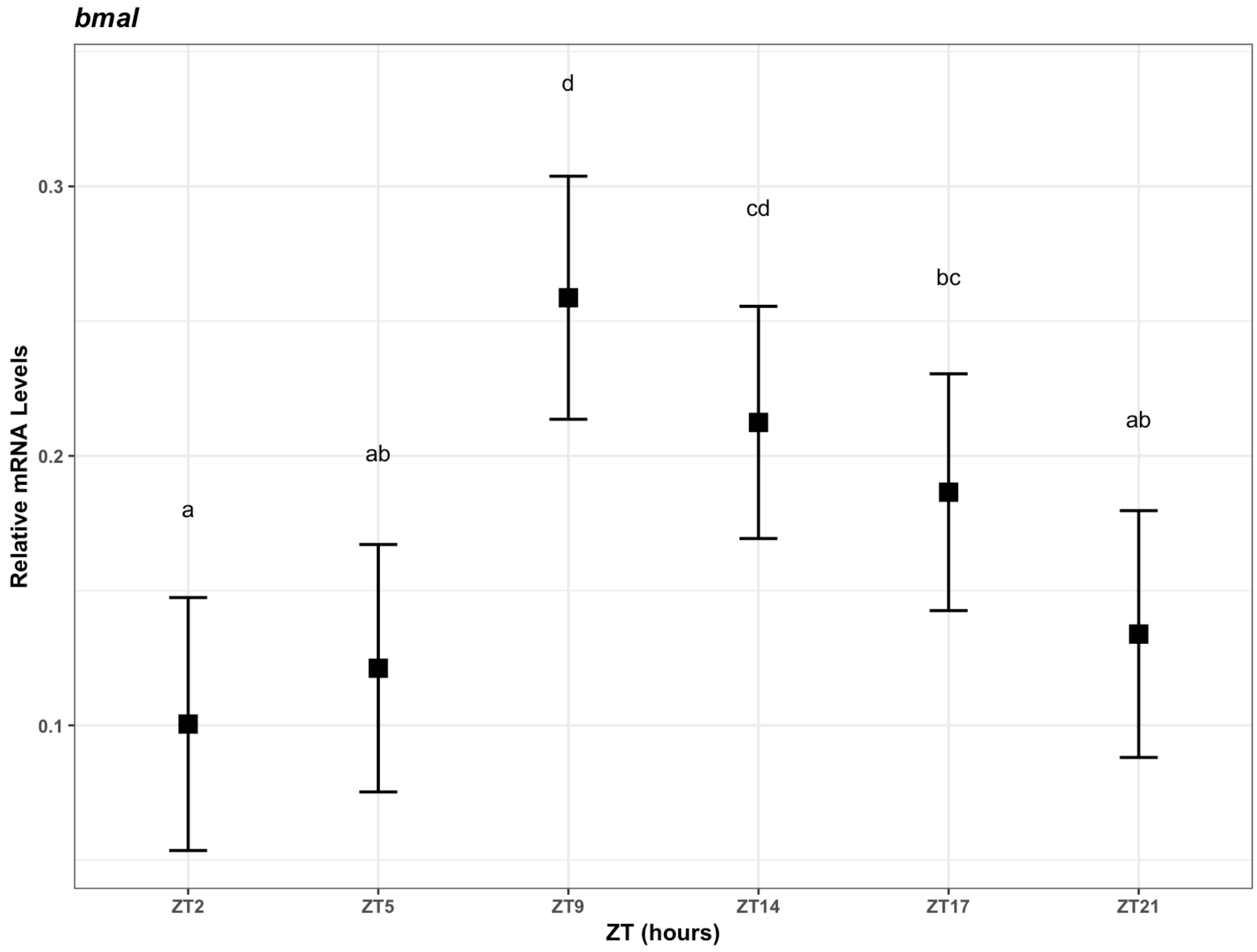
bmal
Relative mRNA Levels
0.3
0.2
0.1
d
cd
bc
ab
a
ab
ZT2
ZT5
ZT9
ZT14
ZT17
ZT21
ZT (hours)

2) per
Evaluation of data set for ANOVA assumptions (Homogeneity of variances and Normality by Histogram of Residuals).
)

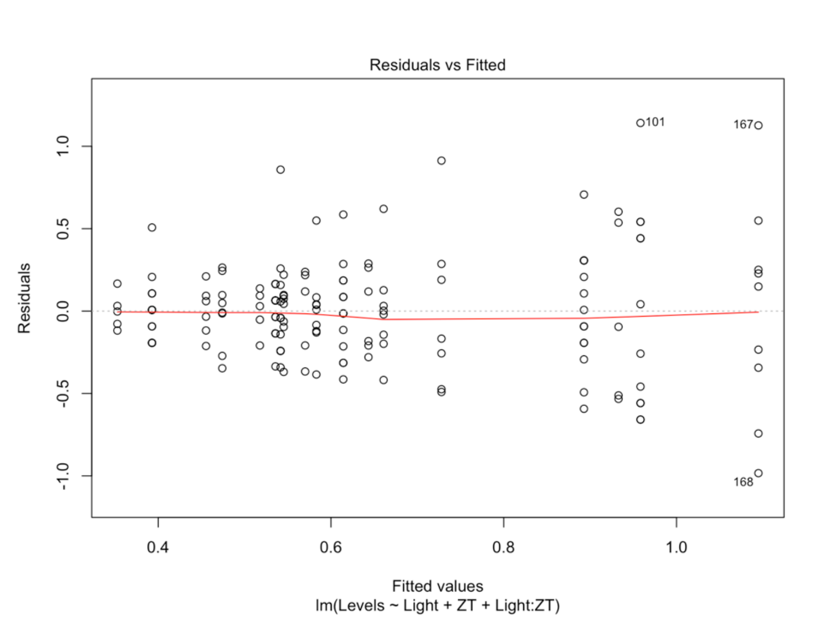

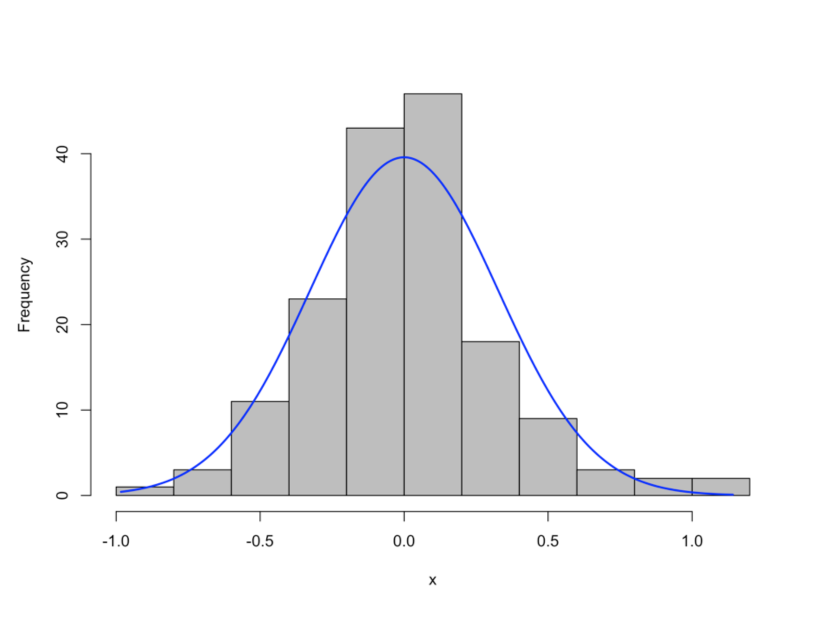

2.1) Two_way ANOVA (Type III)

Response:	Levels				
	Sum Sq	Df	F value	Pr(>F)	
(Intercept)	57.148	1	479.126	2.20E-16	***
Light	0.212	2	0.8871	0.4140844	
ZT	2.709	5	4.5419	0.0007098	***
Light:ZT	3.317	10	2.7811	0.0035836	**
Residuals	17.176	144			

Signif. codes: 0 '***' 0.001 '**' 0.01 '*' 0.05 '.' 0.1 ' ' 1

2.2) Post hoc pairwise (marginal) Tukey analysis for the interaction.

	contrast	ZT	estimate	SE	df	t.ratio	p.value
**1**	Blue - Red	ZT14	-0.50294417	0.1726814	144	-2.91255544	0.011514675
**2**	Blue – white	ZT14	-0.12774208	0.1820222	144	-0.70179399	0.762811545
**3**	Red – white	ZT14	0.37520209	0.1522907	144	2.46372318	0.039385882
**4**	Blue – Red	ZT17	0.10770167	0.1685198	144	0.63910397	0.798798822
**5**	Blue – white	ZT17	-0.01751173	0.1865172	144	-0.09388801	0.995152103
**6**	Red – white	ZT17	-0.12521340	0.1530657	144	-0.81803695	0.692477520
**7**	Blue – Red	ZT2	-0.32272915	0.1799299	144	-1.79363818	0.175339104
**8**	Blue – white	ZT2	-0.15781671	0.2022238	144	-0.78040639	0.715648027
**9**	Red – white	ZT2	0.16491244	0.1598719	144	1.03152849	0.558239688
**10**	Blue - Red	ZT21	0.39120069	0.1838334	144	2.12801754	0.087771631
**11**	Blue – white	ZT21	-0.16194397	0.1926342	144	-0.84068141	0.678398756
**12**	Red – white	ZT21	-0.55314466	0.1522907	144	-3.63216343	0.001129594
**13**	Blue – Red	ZT5	-0.09653653	0.1799299	144	-0.53652300	0.853480923
**14**	Blue – white	ZT5	-0.02757009	0.1968871	144	-0.14002993	0.989248809
**15**	Red – white	ZT5	0.06896645	0.1530657	144	0.45056758	0.894231495
**16**	Blue – Red	ZT9	-0.04007472	0.1799299	144	-0.22272404	0.973029260
**17**	Blue – white	ZT9	-0.12141309	0.1926342	144	-0.63027803	0.803724422
**18**	Red – white	ZT9	-0.08133837	0.1475551	144	-0.55124067	0.846007120

Blue bars correspond to confidence intervals (95%) for the EMMs (Estimated Marginal Means). Red arrows indicate the comparisons among them. If an arrow from one mean overlaps an arrow from another group, the difference is not significant.

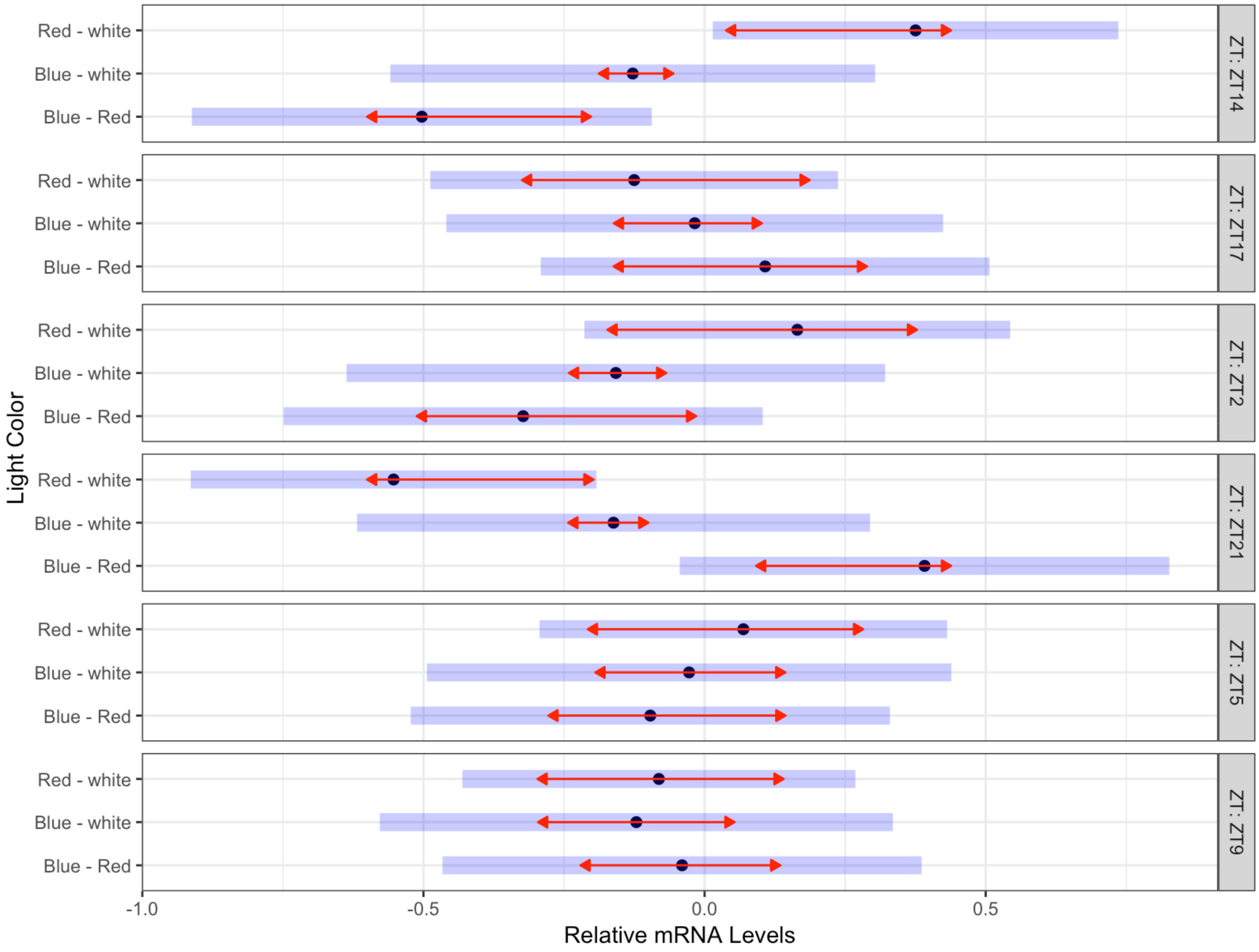

3) tr-cry

Evaluation of data set for ANOVA assumptions (Homogeneity of variances and Normality by Histogram of Residuals).

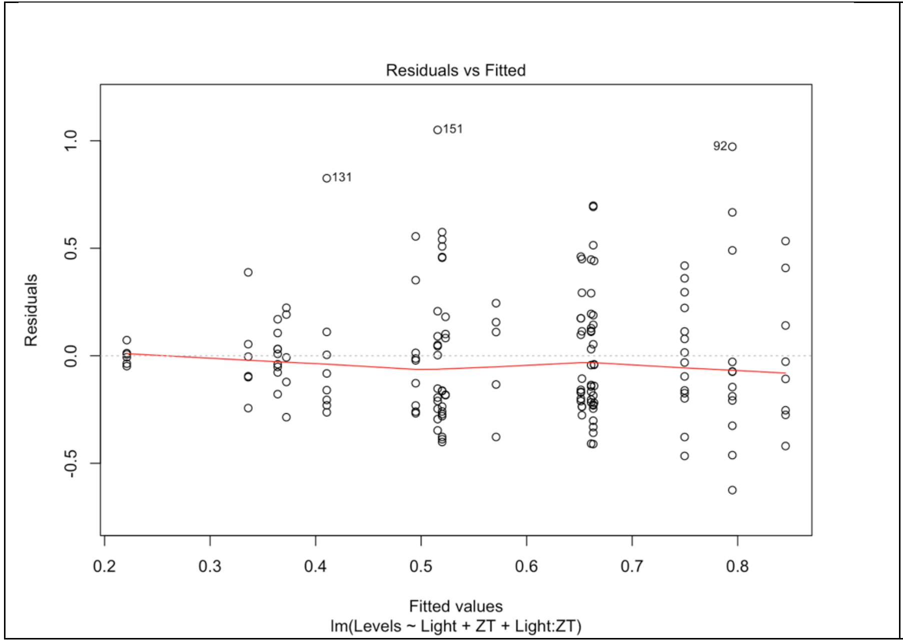

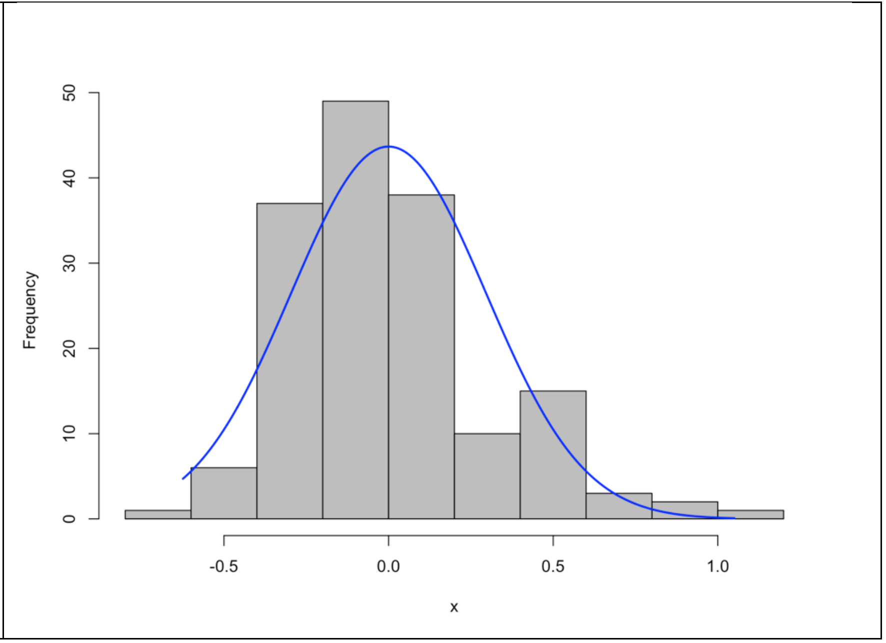

3.1) Two_way ANOVA (Type III)

	Sum SQ	Df	F value	Pr(>F)	
(Intercept)	43.344	1	442.5651	2.20E-16	***
Light	0.952	2	4.8614	0.009056	**
ZT	1.268	5	2.5895	2.83E-02	*
Light:ZT	2.067	10	2.1109	0.027106	*
Residuals	14.103	144			

Signif. codes: 0 ‘***’ 0.001 ‘**’ 0.01 ‘*’ 0.05 ‘.’ 0.1 ‘ ’ 1

3.2) Post hoc pairwise (marginal) Tukey analysis for the interaction.

	contrast	ZT	estimate	SE	df	t.ratio	p.value
**1**	Blue - Red	ZT14	-0.458842232	0.1564747	144	-2.93237278	0.010862436
**2**	Blue - white	ZT14	-0.027924458	0.1649388	144	-0.16930189	0.984324059
**3**	Red - white	ZT14	0.430917774	0.1379977	144	3.12264374	0.006102915
**4**	Blue - Red	ZT17	-0.528654617	0.1527037	144	-3.46196342	0.002029578
**5**	Blue - white	ZT17	-0.189527102	0.1690120	144	-1.12138237	0.502492148
**6**	Red - white	ZT17	0.339127514	0.1387000	144	2.44504298	0.041294206
**7**	Blue - Red	ZT2	-0.092080182	0.1630429	144	-0.56476034	0.839024655
**8**	Blue - white	ZT2	-0.081382920	0.1832444	144	-0.44412218	0.897072270
**9**	Red - white	ZT2	0.010697262	0.1448674	144	0.07384172	0.996998435
**10**	Blue - Red	ZT21	-0.143370021	0.1665801	144	-0.86066732	0.665905743
**11**	Blue - white	ZT21	-0.122550448	0.1745549	144	-0.70207409	0.762647156
**12**	Red - white	ZT21	0.020819573	0.1379977	144	0.15086894	0.987531056
**13**	Blue - Red	ZT5	0.002827343	0.1630429	144	0.01734110	0.999834222
**14**	Blue - white	ZT5	-0.181463647	0.1784086	144	-1.01712365	0.567283243
**15**	Red - white	ZT5	-0.184290990	0.1387000	144	-1.32870196	0.381624403
**16**	Blue – Red=	ZT9	0.003185466	0.1630429	144	0.01953759	0.999789571
**17**	Blue - white	ZT9	-0.128292585	0.1745549	144	-0.73496997	0.743145409
**18**	Red - white	ZT9	-0.131478051	0.1337066	144	-0.98333254	0.588566909

Blue bars correspond to confidence intervals (95%) for the EMMs (Estimated Marginal Means). Red arrows indicate the comparisons among them. If an arrow from one mean overlaps an arrow from another group, the difference is not significant.

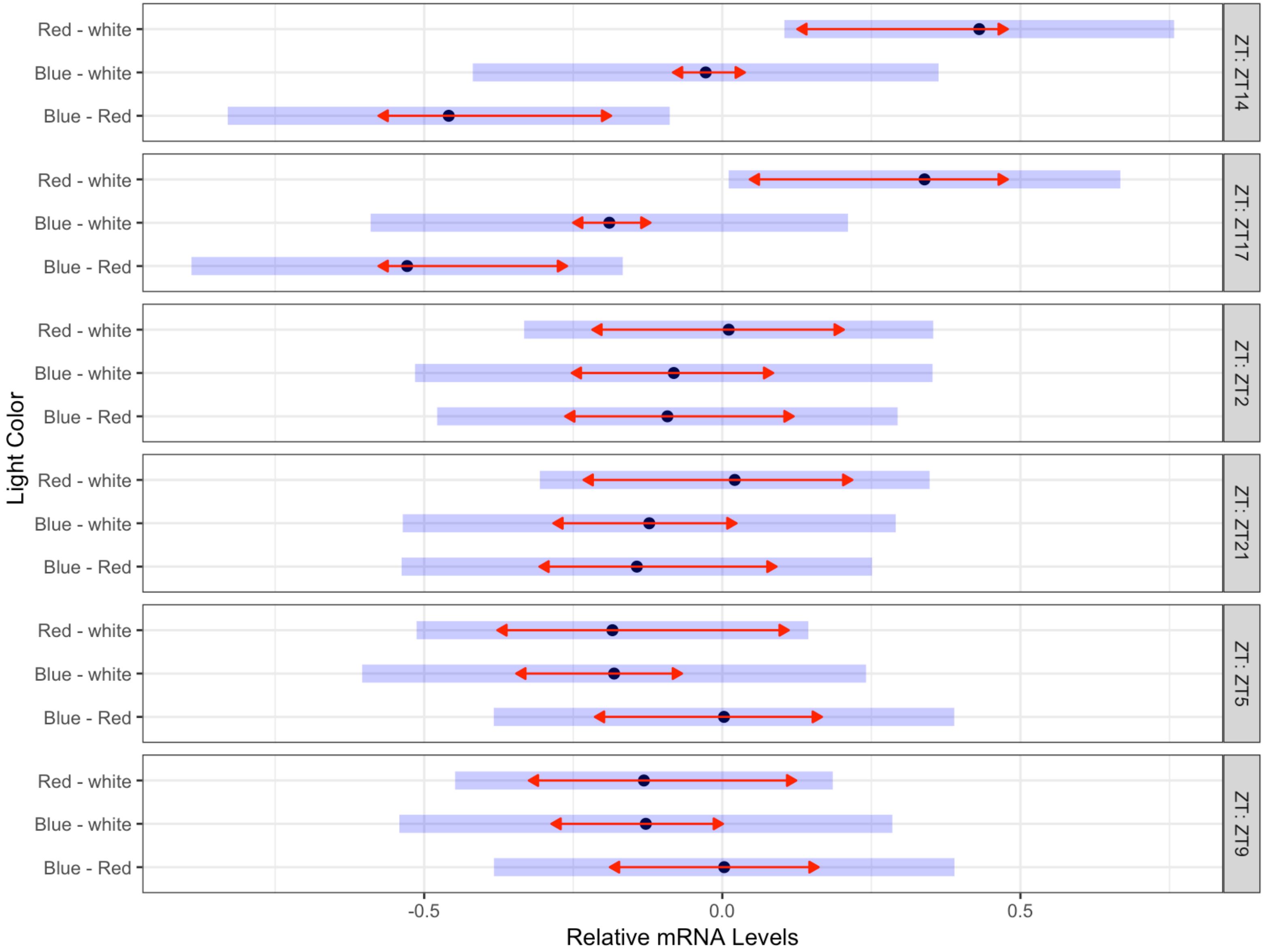

Figure 5D:

Two way repeated sampling ANOVA (Chromatophore size CT2 and CT14 from DD1 to DD4)

**Two-way RM ANOVA**	**Matching: Both factors**					
Assume sphericity?	No					
Alpha	0.05					
**Source of Variation**	**% of total variation**	**P value**	**P value summary**	**Significant?**	**Geisser-Greenhouse's epsilon**	
day of DD	23.26	0.0007	***	Yes	0.6322	
time of subj. day	27.24	0.0016	**	Yes	1	
day of DD x time of subj. day	18.06	0.005	**	Yes	0.4903	
Subject x day of DD	6.69					
Subject x time of subj. day	3.513					
Subject	14.47					
**ANOVA table**	**SS**	**DF**	**MS**	**F (DFn, DFd)**	**P value**	
day of DD	2522	3	840.8	F (1,897, 9,483) = 17,38	P=0,0007	
time of subj. day	2954	1	2954	F (1,000, 5,000) = 38,76	P=0,0016	
day of DD x time of subj. day	1959	3	652.9	F (1,471, 7,355) = 13,34	P=0,0050	
Subject x day of DD	725.5	15	48.36			
Subject x time of subj. day	381	5	76.2			
Subject	1569	5	313.8			
Residual	734.1	15	48.94			
**Difference between column means**						
Mean of CT2	48.44					
Mean of CT14	32.75					
Difference between means	15.69					
SE of difference	2.52					
95% CI of difference	9,211 to 22,17					

Sidak's multiple comparisons test for CT2-CT14 for each DD

**2 way ANOVA multiple comparisons**								
Number of comparisons per family	4							
Alpha	0.05							
**Sidak's multiple comparisons test**	**Mean Diff,**	**95,00% CI of diff,**	**Significant?**	**Summary**	**Adjusted P Value**			
CT2 - CT14								
DD1	36.74	12,26 to 61,22	Yes	**	0.0093			
DD2	11.88	-1,312 to 25,08	No	ns	0.0737			
DD3	11.89	3,083 to 20,69	Yes	*	0.0148			
DD4	2.244	-12,51 to 16,99	No	ns	0.9715			
**Test details**	**Mean 1**	**Mean 2**	**Mean Diff,**	**SE of diff,**	**N1**	**N2**	**t**	**DF**
CT2 - CT14								
DD1	71.47	34.73	36.74	6.458	6	6	5.689	5
DD2	41.64	29.75	11.88	3.48	6	6	3.414	5
DD3	43.46	31.57	11.89	2.322	6	6	5.119	5
DD4	37.2	34.96	2.244	3.89	6	6	0.577	5

Sidak's multiple comparisons test

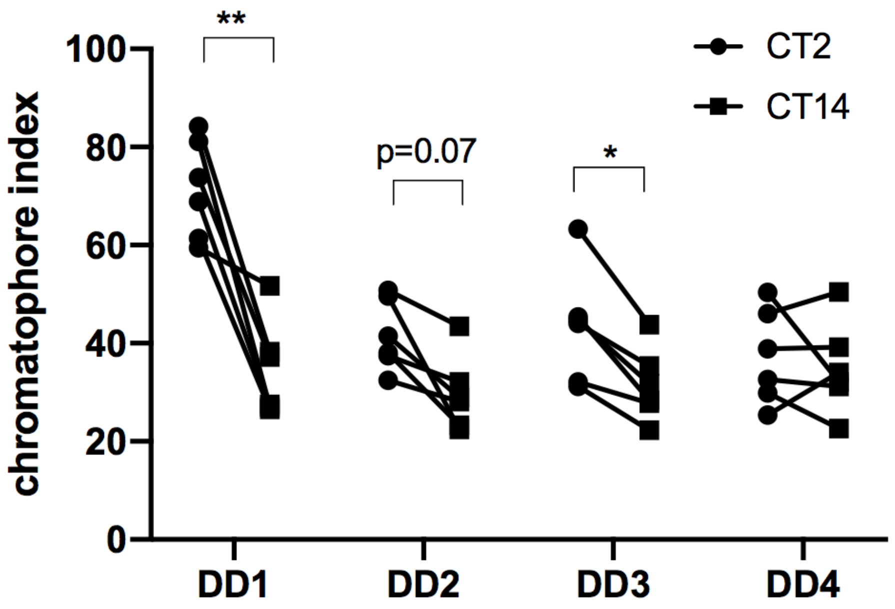

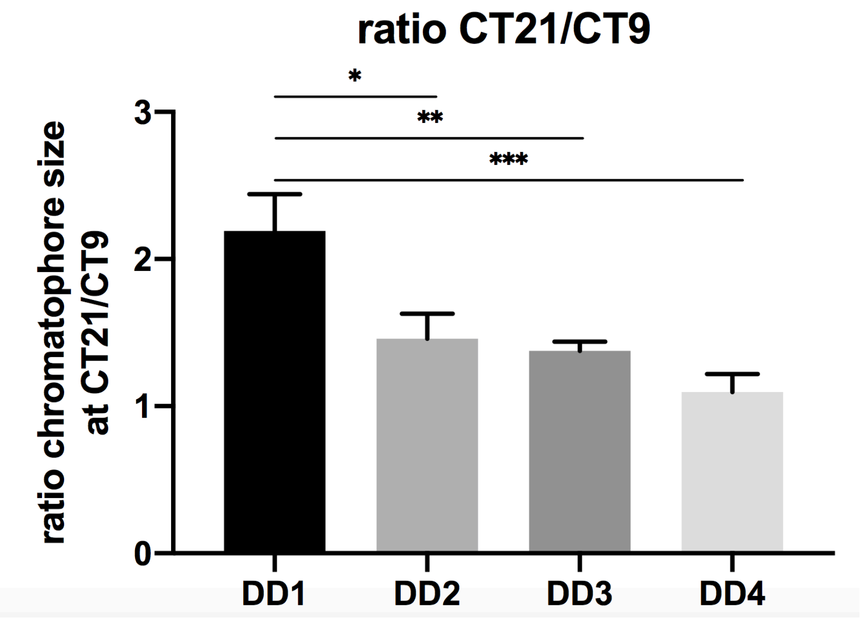

*** p< 0.001
** p<0.01
* p<0.05
